# Hierarchically Soft Porous MOF‐Polymer Monolith for Fast and Large‐Scale Moisture Buffering

**DOI:** 10.1002/advs.202523720

**Published:** 2026-05-08

**Authors:** Guangxin Ma, Xin Zhou, Weiman Li, Ken‐ichi Otake, Susumu Kitagawa, Xiaoze Wang, Mengjie Cao, Linfeng Nie, Ming‐Shui Yao, Yunfa Chen

**Affiliations:** ^1^ State Key Laboratory of Mesoscience and Process Engineering Institute of Process Engineering Chinese Academy of Sciences Beijing P. R. China; ^2^ School of Chemical Engineering University of Chinese Academy of Sciences Beijing P. R. China; ^3^ Institute for Integrated Cell‐Material Sciences Kyoto University Institute for Advanced Study Kyoto University Kyoto Japan

**Keywords:** hierarchical pore structure, humidity control, metal–organic frameworks, synergy, thermal responsiveness

## Abstract

The influence of high indoor humidity on building and equipment safety and human health has led to an urgent demand for the development of high‐performance dehumidification technologies. To simultaneously satisfy the requirements of easy shaping, nontoxicity, and low energy consumption for indoor dehumidification, an effective strategy involving the combination of non‐toxic microporous materials with a shaping matrix equipped with switching functionalities via a synergetic porous interface can be used. This study proposes an in situ interface‐controlled growth method to produce a novel hierarchically porous hydrogel adsorbent for indoor dehumidification by combining metal–organic frameworks (MOFs) with a poly(N‐isopropylacrylamide) (PNIPAM) thermoresponsive hydrogel matrix. The obtained composite hydrogel monolith, ThermoGel‐23, exhibits a moisture uptake capacity of 1.64 g·g^−1^, high adsorption rate, and desorption activation energy as low as 31.0 kJ·mol^−1^, meeting the requirements of indoor adsorption dehumidification. Combining experiments and simulations, we elucidated the transport pathways of water clusters within the hierarchical pores and highlighted the essential role of porous structure and thermal response in enhancing both adsorption and desorption. This work demonstrates the potential of MOF–polymer composites as efficient, scalable desiccants, paving the way for sustainable and energy‐saving humidity control materials.

## Graphical abstract

A soft, hierarchical porous monolith that combines metal–organic frameworks (MOFs) with a thermoresponsive polymer matrix enables rapid, large‐scale moisture buffering. The synergistic interface facilitates high‐capacity water capture and low‐energy release for sustainable indoor dehumidification.

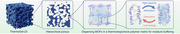

## 1 Introduction

Confined spaces, such as hospital wards, precision manufacturing facilities, aerospace equipment, and facilities used for the storage of valuables, require humidity control [1]. In indoor environments, excessive humidity not only triggers complex chemical reactions between different gases but also causes damage to buildings and equipment. For the condensation dehumidification technology, which is the most widely used method for controlling indoor humidity [2], the cooling and reheating processes fail to separate sensible heat from temperature changes and latent heat from phase changes, resulting in high energy consumption and CO_2_ emissions [3]. The increasing need to lower the costs and increase the efficiency of energy consumption has given rise to an increasing demand for the development of adsorption dehumidification technologies. In the adsorption dehumidification process, desiccants can spontaneously adsorb water molecules and release adsorption heat (an isenthalpic process), enabling independent control of the sensible and latent heats of the water molecules [4].

Various hygroscopic materials, including silica gels, zeolites, and emerging hydrogels, have been used in different dehumidification scenarios. The main components of commercial hygroscopic salts, such as calcium chloride [5] and lithium chloride [6, 7] (contributing about 95%–98% to water uptake [8]), have high regeneration temperatures (200°C and 186°C, respectively), and face the problems of easy leakage and equipment corrosion. Owing to their large surface area, regular porosity, and appropriate binding energy with water molecules [9, 10, 11, 12], metal–organic frameworks (MOFs) have been investigated as emerging hygroscopic materials with high water vapor uptake capacity and low energy consumption [13, 14, 15, 16, 17, 18]. To simultaneously satisfy the requirements of indoor dehumidification applications for easy shaping, non‐toxicity, and low energy consumption, MOFs can be combined with functional shaping materials [19, 20]. Several studies have shown that MOF‐gel materials such as MIL‐160(Al)/PAA‐NH_2_ [21], Ni‐MOF/PDA [22], poly(N‐isopropylacrylamide)/MIL‐101(Cr) (PNIPAM/MIL‐101(Cr)) [23], and MIL‐101(Fe, Cr)@xerogel [24] have excellent hygroscopic properties. However, these composites still face challenges of potential toxicity, unclear water‐uptake/release mechanisms, and/or unsatisfactory adsorption capacity and desorption energy consumption [25]. Therefore, an ideal composite should contain a non‐toxic, highly accessible MOF, a stable porous polymer matrix with additional switching functionalities, and synergistic porous interfaces to form a hierarchically soft porous structure [3, 26].

**FIGURE 1:**
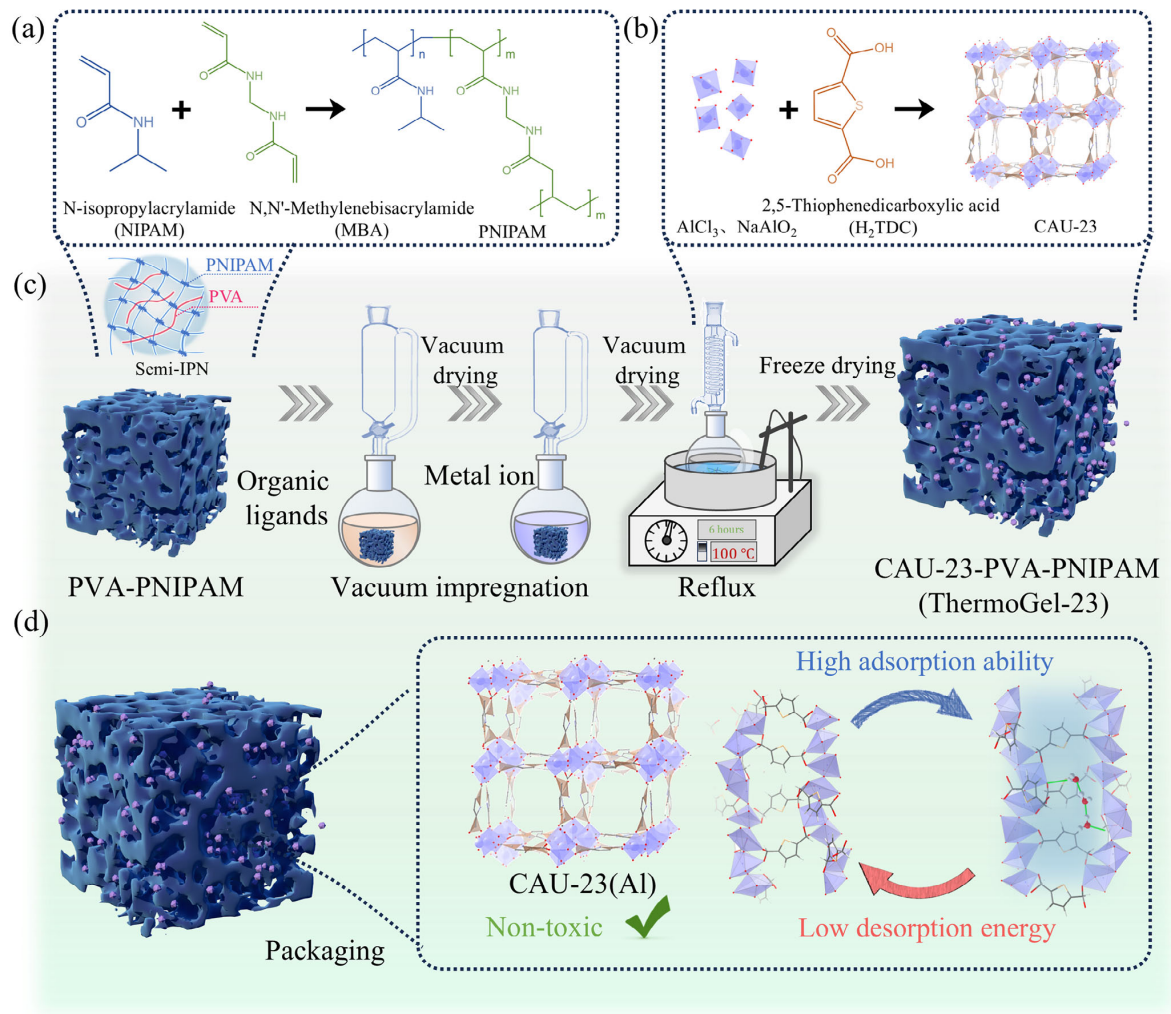
(a) Synthesis of PNIPAM by the chemical cross‐linking method. (b) Synthesis of CAU‐23 by the reflux method. (c) Synthesis of ThermoGel‐23 by the in situ growth method. (d) Advantages of CAU‐23‐PVA‐PNIPAM (ThermoGel‐23) as a desiccant.

In this study, we report an in situ interface‐controlled growth strategy for packaging MOF (CAU‐23) into a thermoresponsive polymer matrix to form a hierarchically soft porous structure that combines the merits of MOFs, polymers, and their soft porous interfaces (Figure 1c and Figure S1). The obtained CAU‐23‐poly(vinyl alcohol)‐poly(N‐isopropylacrylamide) (CAU‐23‐PVA‐PNIPAM) MOF‐gel monolith, termed ThermoGel‐23, not only overcomes the difficulty of shaping encountered for the MOF powder but also creates abundant active sites and fast transport pathways. ThermoGel‐23 responds to temperature changes, changing from hydrophilic to hydrophobic at 37°C, thus readily releasing water [27, 28, 29]. Owing to these merits, ThermoGel‐23 exhibits exceptionally high and rapid water vapor uptake and release. The strategy used to obtain ThermoGel‐23 provides an efficient pathway for rational design of desiccants with high adsorption capacities and low desorption energies.

## 2 Results and Discussion

**FIGURE 2:**
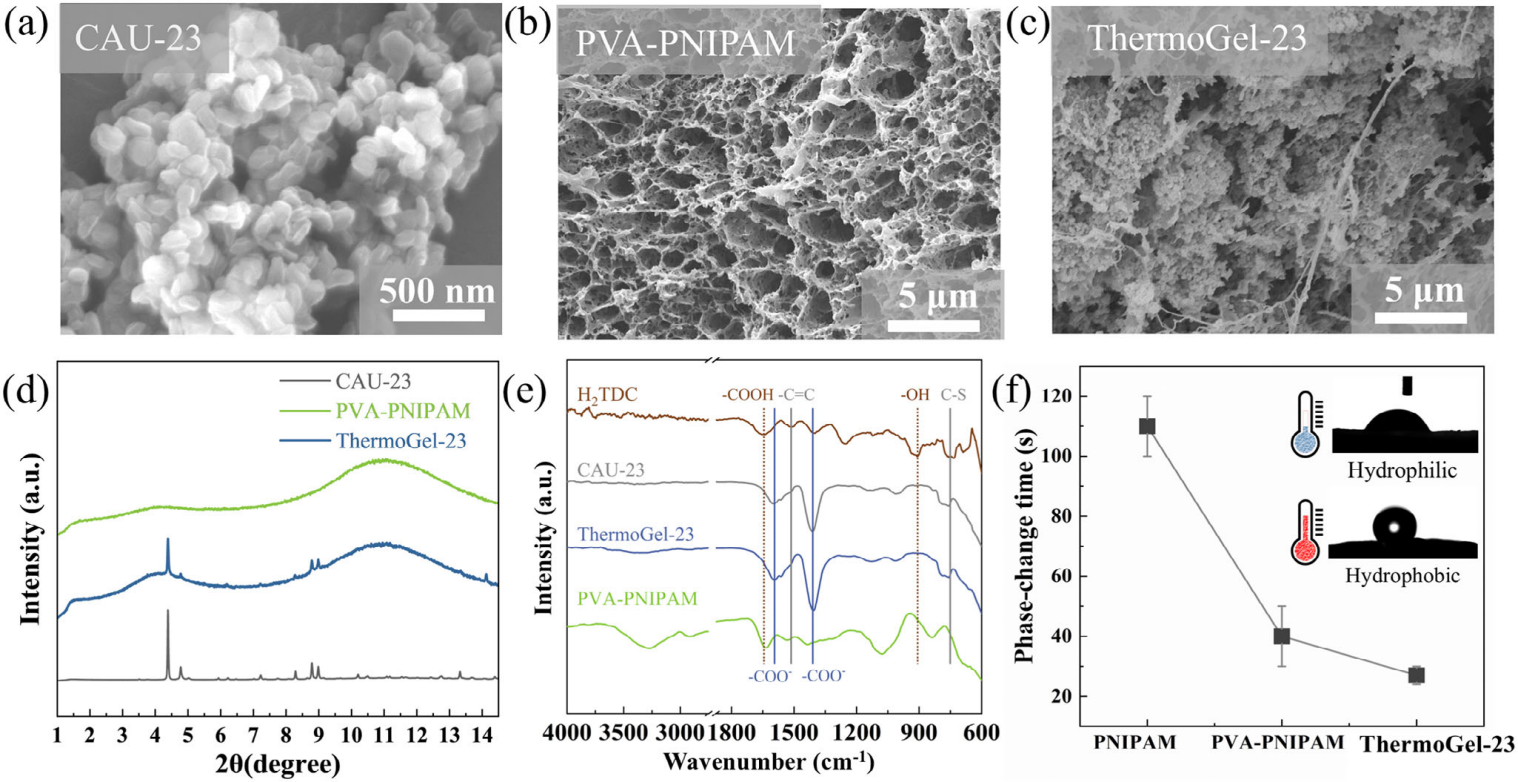
CAU‐23, PVA‐PNIPAM, ThermoGel‐23 (a–c) SEM images obtained by the in situ growth method. (d) XRD pattern collected at beamline 02B2 at Spring‐8, Japan (λ = 0.79962 Å). (e) FTIR spectrum. (f) Hydrogel phase‐change times and water contact angle for PNIPAM, PVA‐PNIPAM, and ThermoGel‐23.

To avoid pore blockage associated with the conventional sol–gel method (see comparison in Figures S2–S5), we developed an in situ interface‐controlled growth strategy. Specifically, ligands were first loaded onto a pre‐prepared PVA‐PNIPAM gel. After vacuum‐drying, metal salt solutions were added to grow the MOF directly within the gel pores (Figure 1c). The morphology of ThermoGel‐23 is shown in Figure 2a–c. The diameter of the CAU‐23 particles in the composite gel prepared using the in situ interface‐controlled growth method was approximately 100 nm. The channels of ThermoGel‐23 obtained by the in situ growth method exhibited a more ordered network structure, and CAU‐23 was evenly distributed within the gel network. This improvement can be attributed to two factors: first, the excellent thermal stability of PVA‐PNIPAM [30], which did not exhibit any significant structural changes before and after the reaction; Second, the shrinkage phase transition of PVA‐PNIPAM during reflux (*T* > lower critical solution temperature (LCST)) effectively confines the organic ligands and metal sources within the gel network, which is attributed to the thermal responsiveness of the PNIPAM components (the phase transition mechanism will be discussed). Scanning electron microscopy (SEM) of the surface and internal cross‐section of ThermoGel‐23 (Figure S6a,b) provides direct evidence supporting internal growth, and energy dispersive spectroscopy (EDS) also shows that the characteristic elements of CAU‐23 are uniformly distributed inside ThermoGel‐23 (Figure S7a,b).

The crystal structure of the ThermoGel‐23 composite was characterized using x‐ray Diffraction (XRD). The results showed that ThermoGel‐23 exhibited obvious CAU‐23 diffraction peaks (Figure 2d). Separately, we confirmed that pure CAU‐23 could be synthesized by adjusting the concentrations of metal and ligands in the precursor solution (Figure S8a). The only by‐product of the CAU‐23 synthesis was residual NaCl (Figure S8b). By contrast, no clear polymer peak was observed in the physical mixture of PVA‐PNIPAM and CAU‐23 (Figure S8c), indicating that the strong XRD patterns of the dominant exposed MOF obscured the XRD patterns of the polymer. In summary, the in situ growth method enables uniform growth of CAU‐23 within the PVA‐PNIPAM channels, rather than just on the surface.

To further investigate the interaction between CAU‐23 and gels, Fourier‐transform infrared (FTIR) spectroscopy analysis was performed, and the results are shown in Figure 2e. The C─OH bending vibration peak (908 cm^−1^) and free carboxylic acid group (1646 cm^−1^) were observed in the H_2_TDC spectrum but not in the spectra of CAU‐23 and ThermoGel‐23. This indicates that the carboxylate ligand was successfully coordinated with aluminum [31]. The peaks of the CAU‐23‐containing gel centered at 1594 and 1411 cm^−1^ can be attributed to the asymmetric and symmetric stretching vibrations of the carboxylate group, and the peaks at 1513 and 755 cm^−1^ are attributed to the C═C and C─S ring vibrations [32]. These results indicate that CAU‐23 was successfully synthesized in the gel matrix.

The pore architecture of the composites was systematically characterized. As shown in Figure S8d, both pristine CAU‐23 and ThermoGel‐23 exhibit type II N_2_ adsorption–desorption isotherms, indicative of microporous structures [33, 34]. In contrast, the blank PVA‐PNIPAM matrix possesses predominantly mesopores and macropores (20–10 000 nm) (Figure S9). The integration of these components results in ThermoGel‐23 possessing a hierarchical micropore‐mesopore‐macropore network, as further visualized in Figure S10a,b, and summarized in Table S3. To quantitatively evaluate the accessibility of the CAU‐23 micropores within this hierarchical network, CO_2_ physisorption at 273 K was employed as a complementary probe. The specific surface area (SSA) of ThermoGel‐23 is 407.89 m^2^·g^−1^. After subtracting the minor contribution from the PVA‐PNIPAM matrix (24.79 m^2^·g^−1^), resulting in an effective accessible MOF fraction of approximately 43.9% (see Section S1.7 for calculation details). This accessible fraction (∼44%) is substantially lower than that of optimized MOF–polymer composites, where accessible MOF fractions of 70%–80% have been demonstrated [35]. The partial retention of microporosity is consistent with the reduced water uptake of ThermoGel‐23 compared to pure CAU‐23 at low relative humidity, indicating some degree of pore blockage. Nonetheless, the retained micropores still contribute to the overall sorption through synergy with the meso‑ and macropore network.

Thermogravimetric analysis (TGA) showed that the thermal decomposition temperatures of PVA‐PNIPAM, CAU‐23, and ThermoGel‐23 are 411.1°C, 461.4°C, and 379.2°C–396.1°C, respectively (Table S4), indicating that the composite possesses good thermal stability (Figure S11a,b). The decrease in the thermal decomposition temperature can be attributed to the enhanced heat transport and smaller particle size of CAU‐23, which accelerated heat conduction in the gel matrix. The initial contact angle of CAU‐23 is approximately 50°, indicating its hydrophilicity (Figure S12). Comparison with PVA‐PNIPAM and ThermoGel‐23 revealed that in situ anchored CAU‐23 did not significantly affect the hydrophilicity of the gel. Moreover, PNIPAM has an anisotropic structure and shows a slow phase transition response (∼110 s) (Figure S9). By contrast, PVA‐PNIPAM with a semi‐interpenetrating network (semi‐IPN) presents a superior structure with more channels and greater isotropy, accelerating the water release process (∼40 s) (Figure 2f) [36]. ThermoGel‐23 demonstrated a faster response than PVA‐PNIPAM owing to the more ordered crystal structure of CAU‐23 that provides higher thermal conductivity [37, 38]. Meanwhile, PNIPAM hydrogels exhibit poor mechanical strength, and their structure is easily damaged by the in situ growth of CAU‐23; therefore, PVA was added to construct a semi‐IPN.

**FIGURE 3:**
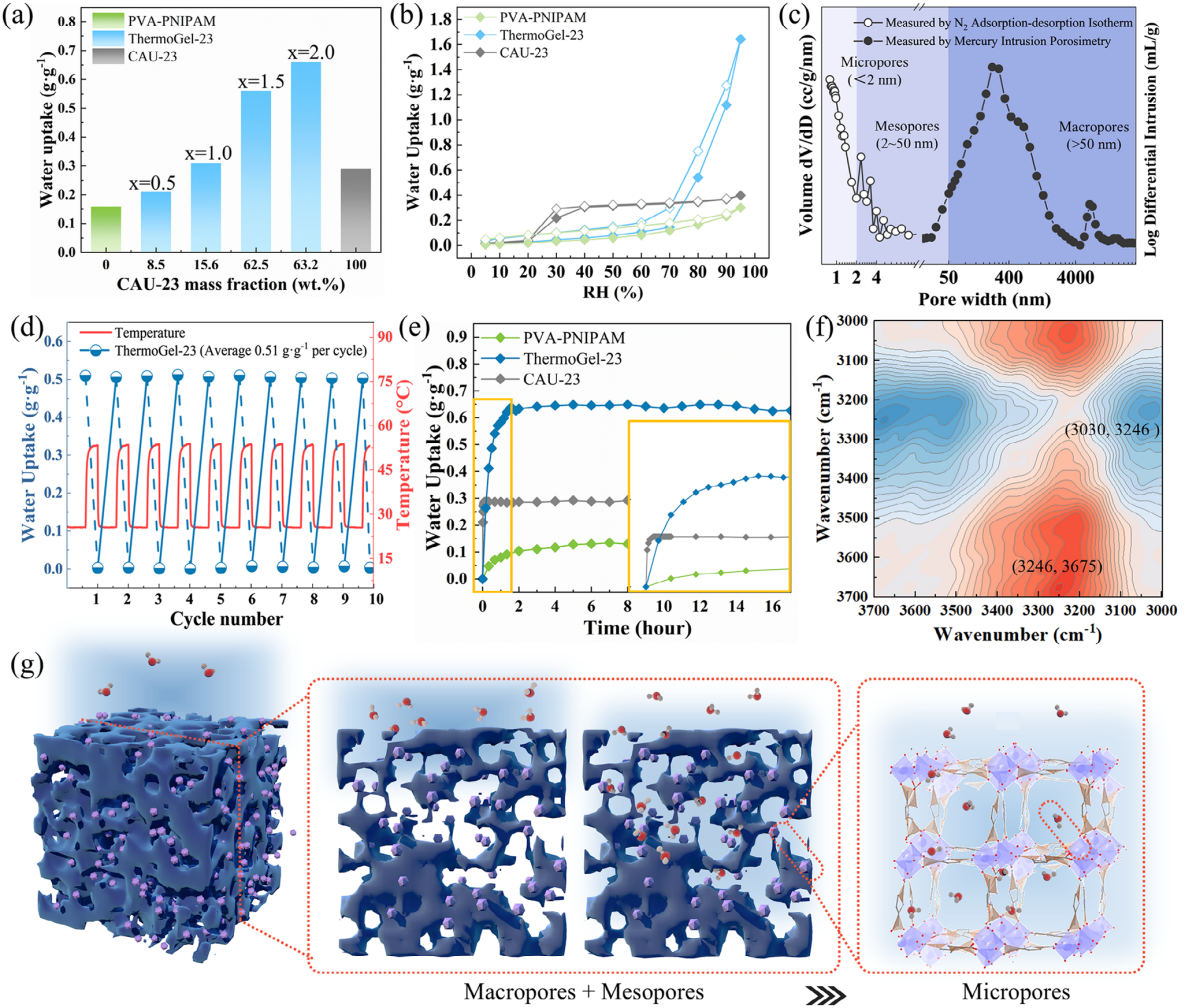
(a) Water uptake of ThermoGel‐23 with different CAU‐23 mass fractions (*T* = 25°C, RH 80%). (b) Water vapor adsorption‐desorption isotherms (*T =* 25°C). (c) Pore size distribution of ThermoGel‐23. (d) The cycling stability of ThermoGel‐23 (*T* = 25°C, RH 80%). (e) Adsorption kinetics of ThermoGel‐23 (*T* = 25°C, RH 80%). (f) 2D‐FTIR correlation asynchronous spectra of ThermoGel‐23 (3700–3000 cm^−1^) and (g) Sorption of hygroscopic hydrogel.

Figure 3a shows that the incorporation of CAU‐23 into PVA‐PNIPAM facilitates water uptake. The ThermoGel‐23 with 56.3% CAU‐23 loading prepared using high ligand and metal concentrations exhibited a water uptake of 0.65 g·g^−1^ (Relative humidity (RH) 80%), which is significantly higher than that of pure CAU‐23 powder (0.30 g·g^−1^). Therefore, our subsequent investigations focused on ThermoGel‐23 prepared with high ligand and metal concentrations (2.0 ThermoGel‐23 in the Supporting Information). The water vapor adsorption‐desorption isotherms (Figure 3b) show that the water absorption rates of CAU‐23, PVA‐PNIPAM and ThermoGel‐23 are 0.22, 0.03 and 0.04 g·g^−1^ (RH 30%), 0.32, 0.08 and 0.11 g·g^−1^ (RH 60%), and 0.40, 0.30 and 1.64 g·g^−1^ (RH 95%), respectively. Among them, CAU‐23 showed the most significant increase in moisture absorption under low humidity conditions, while ThermoGel‐23 mainly showed an increase under high humidity conditions (the water uptake from 60% to 95% RH is ∼1.54 g·g^−1^) (Figure S13a). Inductiveky coupled plasma optical emission spectrometry (ICP‐OES) quantitative analysis showed that the residual NaCl content was 9.54 wt.%.

To explicitly deconvolute the influence of this residual NaCl on the overall performance, a control sample (ThermoGel‐23 without NaCl) was prepared via thorough washing, resulting in a low NaCl content of 0.007–0.009 wt.% as confirmed by ICP‐OES. The moisture uptake of this control sample at 95% RH was measured to be 1.27 g·g^−1^ (Figure S13b), confirming that the residual NaCl contributes approximately ∼23% of the total capacity under these conditions. Analysis of the contributions of each component (Figure S13c,d) indicates that the high water uptake of ThermoGel‐23 is due to the combined effect of three factors: (i) the accessible CAU‐23 micropores and the hydrophilicity of the polymer; (ii) capillary condensation in the hierarchical pore network; and (iii) the hygroscopic effect of residual NaCl.

ThermoGel‐23 exhibited excellent cyclic stability, retaining >94% of its initial capacity after 10 adsorption‐desorption cycles (Figure 3d and Figure S14a–c). Furthermore, after three months of aging under environmental conditions, ThermoGel‐23 maintained its original water vapor adsorption capacity (Figure S14d). This improvement was evident in high‐humidity environments (RH>70%). According to Kelvin's equation, the pore size range for capillary condensation is approximately 2–50 nm [39], whereas the hierarchical pore structure significantly increases the amount of capillary condensation adsorption in high‐humidity environments [23]. In low to medium humidity environments, water molecules combine with the hydrophilic sites, leading to the adsorption of a small amount of water. This hypothesis is further discussed below.

In addition to the equilibrium adsorption performance, the adsorption rate of the material should also be considered. The adsorption process of the ThermoGel‐23 gel was completed in approximately 2 h (Figure 3e), and thus is much faster than that of PVA‐PNIPAM (6 h). To explore the effect of pore structure on the adsorption rate, the adsorption kinetics were fitted and analyzed (Figure S15) [40, 41]. Pseudo‐first‐order adsorption was modeled according to the Lagergren model, which is commonly used to describe the process of water vapor entering the interior of pores from the boundary film (Equation S1). A pseudo‐second‐order adsorption kinetic model was used to describe the diffusion behavior of water vapor in the pores (Equation S2). The results of the adsorption kinetics fitting (Table S5) for the gel show that the construction of a hierarchical porous structure can accelerate moisture adsorption [42, 43]. Two‐dimensional correlation spectroscopy (2DCS) was used to further analyze the adsorption process. The synchronized spectrum indicated that the O─H stretching vibration had a strong response to the water adsorption process (Figure S16). The positive peak (orange on the lower side, *ψ* (3246, 3675)) and the negative peak (blue on the right‐hand side, *ψ* (3030, 3246)) in the asynchronous spectrum [44] indicate the presence of three types of water in ThermoGel‐23 (Figure 3f) [45]: intermediate water that is weakly bound by hydrogen bonds (3675 cm^−1^), water molecule clusters (3246 cm^−1^), and bound water held by strong hydrogen bonds (3030 cm^−1^) [46]. According to Noda's theory [47], the diffusion path of water from the atmosphere to the hydrogel can be described as water molecule clusters → bound water → (intermediate water and free water) corresponding to 3246 → 3030 → 3675 cm^−1^, which is consistent with results obtained in previous studies [48, 49].

**FIGURE 4:**
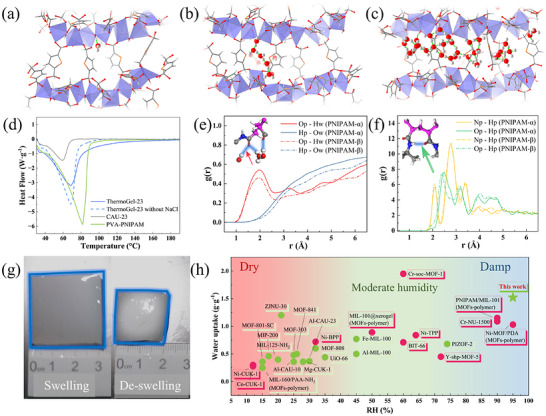
GCMC simulated adsorption process and radial distribution functions of water network in ThermoGel‐23: (a–c) snapshot of water molecules distribution in CAU‐23 microporous cage; (d) DSC spectrum of water‐containing hydrogels. (e) Radial distribution functions of H_2_O and PNIPAM‐α (*T* < LCST) or PNIPAM‐β (*T* > LCST). (f) Interatomic radial distribution functions of PNIPAM‐α and PNIPAM‐β. (g) Photographs of ThermoGel‐23 in the swelling state (*T* < LCST, hydrophilic) and in the de‐swelling state (*T* > LCST, hydrophobic). (h) Comparison of water uptake of MOF and hydrogel materials (green dots: non‐toxic, red dots: toxic).

Grand Canonical Monte Carlo (GCMC) simulations were used to investigate the adsorption process of the water clusters as they traversed the hierarchical pores of ThermoGel‐23. Simulation snapshots revealed that adsorption primarily occurred within the microporous cages of CAU‐23 (Figure 4a–c). Initially, oxygen atoms (O_w_) (here the first symbol denotes the element and the second indicates its counterpart) of H_2_O formed hydrogen bonds with metal hydroxyl, occupying the primary adsorption sites (Figure 4b). As the pressure ratio P/P_0_ increased, water molecules began to establish hydrogen bond networks with the previously adsorbed water molecules, completely filling the pores (Figure 4c). This behavior can be attributed to the stronger interactions between O_w_ and H_w_ within water molecule clusters, compared to the interactions between Ow and H_µOH_ (CAU‐23), as deduced from the examination of the radial distribution functions (RDF) (Figure S17). Specifically, the characteristic distance between H_µOH_ (CAU‐23) and O_w_ is 2.74 Å, while the distance between H_w_ and O_w_ is 1.82 Å. Consequently, the gradual increase in water uptake of CAU‐23 can be attributed to the filling of micropores.

To further elucidate the interactions between the components of the hierarchical gel, a unit cell was constructed based on the feed ratio, revealing the interfacial bonding mechanisms between the polymer chains and CAU‐23. In the semi‐IPN network, the extended PVA chains formed strong hydrogen bonds with the PNIPAM gel network, leading to significant entanglement. The hydroxyl of PVA exhibits the strongest interaction with the amide of PNIPAM [30, 50], with a characteristic H_µOH_ (PVA)‐O_µc = o_ (PNIPAM) distance of 1.89 Å (Figure S18a). MOF generally forms strong interactions with polymers, as indicated by their peak positions in the RDF [23]. The nature of these interactions can also be inferred from the positions of the RDF peaks. The hydroxyl groups of the AlO_6_ polyhedron in CAU‐23 form a strong hydrogen bond with the amino group of PNIPAM, with a characteristic H_µOH_(CAU‐23)‐O_µc = o_ (PNIPAM) distance of 1.58–1.72 Å (Figure S18b) and a robust hydrogen bond with the hydroxyl hydrogen of PVA, with a characteristic H_µOH_(CAU‐23)‐O_µOH_ (PVA) distance of 1.88–1.98 Å (Figure S18c). The strong interactions between CAU‐23, PVA, and PNIPAM enhanced the overall strength and stability of the composite.

Based on the combined experimental and simulation evidence, we propose a multi‐modal adsorption mechanism for ThermoGel‐23: (I) Interface diffusion: water molecule clusters pass through the macropores at the boundary of the hydrogel [51]. (II) Water transport: driven by pressure difference, water clusters are delivered to mesopores and accessible micropores. (III) Low‐to‐moderate RH regime (RH < 60%): accessible CAU‐23 micropores and hydrophilic groups on the polymer network cooperatively bind water molecules, as supported by GCMC simulations of micropore filling. (IV) High RH regime (RH > 60%): capillary condensation in the meso‐ and macropores becomes dominant, further amplified by PNIPAM swelling and the osmotic contribution of residual NaCl. This multi‐mode synergy accounts for the sharp capacity increase above 70% RH and the excellent overall performance.

The desorption ability of water molecules from adsorbents can be characterized by their desorption activation energies. According to the weight loss data of PVA‐PNIPAM and CAU‐23 (Table S6 and Figure S19), the first weight loss stage of ThermoGel‐23 corresponded to surface water evaporation and internal water loss, decomposition of the polymer chain occurred in the second stage, and the third stage involved the collapse of CAU‐23 through thermal decomposition (Figure S20). The desorption activation energy was calculated using the Kissinger equation [52] by fitting the Derivative Thermogravimetry (DTG) desorption peak temperatures at various heating rates (Figure S21 and Table S7). The apparent desorption activation energy of ThermoGel‐23 was 31.0 kJ·mol^−1^. To further investigate the regeneration energy, Differential Scanning Calorimetry (DSC) was employed to measure the enthalpy change associated with water release (Figure 4d and Table S8). The apparent desorption enthalpy of the ThermoGel‐23 composite was 24.9 kJ·mol^−1^. To deconvolute the contributions from the individual components and the residual salt, control experiments were performed. The pure CAU‐23 powder exhibited a low desorption enthalpy of 23.5 kJ·mol^−1^, confirming its low regeneration energy demand. The fact that the composite's enthalpy is lower than that of the polymer matrix (31.5 kJ·mol^−1^) alone indicates that incorporating CAU‐23 reduces the overall energy penalty for dehydration. Comparing ThermoGel‐23 (24.9 kJ·mol^−1^) and ThermoGel‐23 without NaCl (27.5 kJ·mol^−1^), it was found that the presence of NaCl had little effect on the enthalpy. Therefore, the easy release of water by ThermoGel‐23 is mainly attributed to the low energy consumption of CAU‐23 and the thermal response conversion of the hydrogel [53]. It should be noted that the apparent desorption enthalpy (24.9 kJ·mol^−1^) and Kissinger activation energy (31.0 kJ·mol^−1^) reflect the intrinsic energetics of water release from the fully hydrated state under temperature‐swing conditions. These metrics are useful for comparing material‐level properties but do not directly translate to the practical regeneration energy per mass of water removed in a device, which depends on sensible heating, heat recovery, and mass transfer dynamics. Nonetheless, the composite exhibits a lower apparent desorption enthalpy than the polymer matrix alone and compares favorably with many reported desiccants, suggesting an intrinsically facile water release that may be exploited in energy‐efficient system design.

To further analyze the low activation energy of desorption and verify the phase‐change mechanism, molecular dynamics simulations were conducted to simulate the swelling state at temperatures below LCST (*T* < LCST) and the deswelling state at temperatures above the LCST (*T* > LCST) of the ThermoGel‐23 polymer network. The formation of PNIPAM‐H_2_O hydrogen bonds competes with the formation of H_2_O─H_2_O hydrogen bonds [54]. Therefore, a polymer swelling model was constructed (Figure S22), and the changes in hydrogen bonds with increasing temperature were analyzed. The results show that the characteristic hydrogen bond distance (1.98 Å) increased when the temperature exceeded the LCST (Figure 4e). This is because an increase in the temperature disrupts the hydrogen bonds between the amide groups of PNIPAM and the water molecules [55, 56]. Correspondingly, the strength of hydrogen bonds in PNIPAM increased, as indicated by the decrease of the characteristic distance from 2.50 to 2.38 Å (Figure 4f). This suggests that PNIPAM molecular chains, which are near‐linear in their swollen state, curl locally at higher temperatures, and the hydrophobic isopropyl groups at the chain ends wrap around the amide groups, causing steric hindrance and shrinking the overall molecular network (Figure S23) [54]. The macroscopic swelling‐dehydration transition (Figure 4g), accompanied by the change from hydrophilic to hydrophobic surface (Figure 2f) and the phase transition peak observed in the DSC curves (Figure 4d) directly corresponds to the polymer chain reorganization mechanism proposed by MD simulation. Moreover, the energy consumption required for conventional hydrogel regeneration includes both the sensible heat of the temperature change and the latent heat of the phase change [57]. After absorbing moisture in a saturated humid environment, the swollen thermosensitive gel reached its LCST and underwent macroscopic shrinkage, further reducing the energy consumption associated with the desorption process. Compared with the previously reported CaCl_2_‐PVA‐PNIPAM [58] (Table S7), ThermoGel‐23 shows a higher moisture adsorption rate and a lower desorption temperature. The high adsorption rate is due to the hierarchical pore structure [59], while the low desorption temperature is due to the easy release of the water molecules by the hygroscopic CAU‐23 [60]. Comparison to previous reports showed that ThermoGel‐23 outperforms the existing MOF‐polymer non‐toxic hygroscopic agents in high‐humidity environments (Figure 4h and Table S9), directly reflecting the advantages of the use of CAU‐23 as the hygroscopic component.

## 3 Conclusion

In summary, we synthesized a ThermoGel‐23 composite hydrogel using an in situ interface‐controlled growth method to combine the high hygroscopicity of non‐toxic CAU‐23 with the structural flexibility and thermal responsiveness of a PVA‐PNIPAM hydrogel. This composite demonstrates superior water uptake performance (1.64 g·g^−1^ at 25°C and 95% RH), high adsorption rate, and readily releasable water (31.0 kJ·mol^−1^). The composite's high specific surface area (407.89 m^2^·g^−1^), together with its hierarchical pore structure, residual NaCl and synergistic MOF‐polymer interface, contributes to the enhanced moisture adsorption and fast transport kinetics. The semi‐IPN hydrogel network improved the mechanical stability and isotropy. Combined with the experimental water uptake and desorption results, GCMC simulations revealed the initial micropore filling behavior, while two‐dimensional FTIR and kinetic analyses highlighted the multimodal characteristics of the entire process, which, along with gel swelling and the salt effect, drive its excellent high‐humidity performance. These findings underscore the potential of MOF‐polymer composites for the rapid, large‐scale dehumidification in indoor environments, paving the way for further innovation in the development of energy‐efficient and sustainable materials. Future work will focus on scaling up the synthesis to produce large‐scale, mechanically robust monolithic materials for practical dehumidification systems, and evaluating long‐term cycling stability and performance in the presence of conditions including volatile organic compounds and dust.

## Funding

The research was financially supported by the research fund of State Key Laboratory of Mesoscience and Process Engineering (MESO‐24‐02 and MESO‐23‐A06) and Foundation for Innovative Research Groups of the National Natural Science Foundation of China (No. 22421003, 22494633, 22571304, and 2023YFC3708404‐03).

## Conflicts of Interest

The authors declare no conflicts of interest.

## Supporting information




**Supporting File**: advs75599‐sup‐0001‐SuppMat.pdf.

## Data Availability

The data that support the findings of this study are available from the corresponding author upon reasonable request.
