# Supplementary material for: Hierarchically Soft Porous MOF‐Polymer Monolith for Fast and Large‐Scale Moisture Buffering
Source: Adv Sci (Weinh). 2026 May 8;13(43):e23720. doi: 10.1002/advs.202523720 (PMC13335909; doi:10.1002/advs.202523720)
Supplement: Supplementary file 1 — Supporting File: advs75599‐sup‐0001‐SuppMat.pdf. [file ADVS-13-e23720-s001.pdf]

Hierarchically Soft Porous MOF-Polymer Monolith for Fast and Large-Scale Moisture Buffering

Guangxin Ma, Xin Zhou, Weiman Li, Ken-ichi Otake, Susumu Kitagawa, Xiaoze Wang, Mengjie Cao, Linfeng Nie, Ming-Shui Yao,* and Yunfa Chen**

G. Ma, X. Zhou, W. Li, Xi. Wang, M. Cao, L. Nie, M.-S. Yao, Y. Chen
State Key Laboratory of Mesoscience and Process Engineering
Institute of Process Engineering, Chinese Academy of Sciences
Beijing 100190, P. R. China
E-mail: wmli@ipe.ac.cn, msyao@ipe.ac.cn, chenyf@ipe.ac.cn

K.-i. Otake, S. Kitagawa
Institute for Integrated Cell-Material Sciences, Kyoto University Institute for Advanced Study
Kyoto University
Kyoto 606-8501, Japan

X. Zhou, Dr. W. Li, M. Cao, L. Nie, M.-S. Yao, Y. Chen
School of Chemical Engineering
University of Chinese Academy of Sciences
Beijing 100190, P. R. China

Contents

1. Experiment 5
1.1 Materials 5
1.2 Synthesis method 5
1.2.1 Synthesis of CAU-23 5
1.2.2 Synthesis of PVA-PNIPAM 5
1.2.3 Synthesis of ThermoGel-23 6
1.3 Characterizations and Measurements 6
1.4 Two-dimensional correlation spectroscopy 7
1.5 Adsorption kinetics fitting 7
1.6 The desorption activation energy by Kissinger equation 8
1.7 Calculation of the accessible MOF fraction 8
2. Supporting Tables 10
Table S1. Raw materials and Chemical reagents information 10
Table S2. Sample precursor metal ion and ligand concentration 10
Table S3. Specific surface area, pore volume and diameter of composite gels 11
Table S4. Thermal decomposition temperature of hydrogel by the tangent extrapolation method 11
Table S5 Hydrogel adsorption pseudo-first-order and pseudo-second-order kinetic model equation parameters 11
Table S6 Details of the weight loss stage of hydrogel 12
Table S7 Desorption peak temperatures (T_d) and desorption activated energies (E_d) of hydrogel with different heating rates 12
Table S8 The apparent desorption enthalpies of hydrogels during water escape by differential scanning calorimetry (DSC). 12
Table S9 MOF structural parameters and hygroscopic properties 12
3. Supporting Figures 15
Figure S1 Synthesis of PVA-PNIPAM by chemical cross-linking. 15

Figure S2 Synthesis of CAU-23-PVA-PNIPAM-sg by sol-gel method without interface control........15

Figure S3 SEM images of CAU-23-PVA-PNIPAM-sg in sol-gel method: (a) PVA-PNIPAM-sg, (b) 5 wt.% CAU-23-PVA-PNIPAM-sg, (c) 10 wt.% CAU-23-PVA-PNIPAM-sg, (d) 20 wt.% CAU-23-PVA-PNIPAM-sg, (e) 30 wt.% CAU-23-PVA-PNIPAM-sg, (f) 40 wt.% CAU-23-PVA-PNIPAM-sg. 15

Figure S4 CAU-23-PVA-PNIPAM-sg water uptake with different CAU-23 mass fractions by sol-gel method (T=25 °C, RH 80%). 16

Figure S5 CAU-23-PVA-PNIPAM-sg prepared by sol-gel method with different CAU-23 mass fractions. 16

Figure S6 (a) SEM image of ThermoGel-23 surface. (b) SEM image of the internal cross-section of ThermoGel-23. 17

Figure S7 (a) Energy spectrum images of CAU-23. (b) Energy spectrum images of ThermoGel-23. 17

Figure S8 (a) XRD patterns of CAU-23 synthesized from precursor liquids with different concentrations. (b) Crystallization of the supernatant after CAU-23 reflux. (c) ThermoGel-23 physically mixed with different mass ratios. (d) N_2 adsorption and desorption curve. 18

Figure S9 SEM images of PNIPAM. (a) Section view. (b) Plan view........18

Figure S10 (a) Carbon dioxide adsorption curves for micropore analysis. (b) pore size distribution. 19

Figure S11 (a) TG and (b) DTG curves of hydrogels........19

Figure S12 water contact angle and wetting optical images. 19

Figure S13 (a) Water uptake of hydrogels across different humidity ranges. (b) Water vapor adsorption isotherms of ThermoGel-23 without NaCl via thorough washing (*T*= 25 °C). (c) Water vapor adsorption isotherms of components (*T*= 25 °C). (d) Stacked bar graphs of the theoretical water uptake contribution of components (*T*= 25 °C)........20

Figure S14 (a) SEM images of ThermoGel-23 in its initial state. (b) SEM images of ThermoGel-23 after ten adsorption-desorption cycles. (c) TG curves of in its initial state and after ten adsorption-desorption cycles (d) Water vapor adsorption-desorption isotherms of ThermoGel-23 in its initial state and after three months of aging (natural humidity fluctuations). 21

Figure S15 Hydrogel adsorption kinetic equation fitting curves. (a) pseudo-first-order, (b) pseudo-second-order 21

Figure S16 The 2D-FTIR correlation synchronous spectra of ThermoGel-23 (3700-3000 cm^{-1}) 22

Figure S17 (a) Radial distribution functions of the water network. (b) Radial distribution functions between atoms of H_2O and CAU-23 22

Figure S18 (a) radial distribution functions between atoms of PVA and PNIPAM. (b) radial distribution functions between atoms of CAU-23 and PNIPAM And (c) radial distribution functions between atoms of CAU-23 and PVA. 23

Figure S19 (a) The TG and DTG curve of PVA-PNIPAM, (b) The TG and DTG curve of CAU-23 23

Figure S20 The TG and DTG curve of ThermoGel-23 24

Figure S21 ThermoGel-23 desorption activation energy Kissinger equation fitting results. 24

Figure S22 Swelling model of ThermoGel-23 gel polymer chain after molecular dynamics calculation. (a) PNIPAM-α (T< LCST), (b) PNIPAM-β (T > LCST). 25

Figure S23 Schematic diagram of polymer network deswelling 25

4. References 26

1. Experiment

1.1 Materials

Poly(vinyl alcohol)-1799 (PVA, alcoholysis degree: 99%, Mn: 170000), N-Isopropylacrylamide (NIPAM), N,N'-methylenebis(acrylamide) (MBA), N,N,N',N'-tetramethylethylenediamine (TEMED), Methyl alcohol (CH_3OH), 2,5-Thiophenedicarboxylic acid (H_2TDC), Sodium aluminate ($NaAlO_2$) and Aluminum chloride ($AlCl_3$) were purchased from Shanghai Macklin Biochemical Co. Ltd. Sodium hydroxide (NaOH) was purchased from Tianjin Damao Chemicals Reagent Factory. And ammonium persulphate (APS) was purchased from Shanghai Aladdin Biochemical Technology Co. Ltd. Ultrapure water (Millpor 18.2 MΩ·cm) was used throughout all experiments.

All chemical agents used in the experiment were listed below and purchased from commercial sources without further purification (Table S1).

1.2 Synthesis method

1.2.1 Synthesis of CAU-23

CAU-23 was synthesized by reflux method[1]. A clear Na_2TDC ligand solution is obtained by mixing 4.30 g H_2TDC with 2.0 g NaOH in 100 ml deionized water. 18.75 ml 1M $AlCl_3$ and 12.5ml 0.5M $NaAlO_2$ metal salt solution was added to Na_2TDC ligand solution. The concentrations of Na_2TDC, $AlCl_3$, and $NaAlO_2$ in precursor solution were 0.190 M, 0.143 M, and 0.048 M respectively. The suspension was stirred at reflux for 6 hours. After cooling, the precipitate was dispersed in water and centrifuged for three times. CAU-23 powder was obtained from the resulting white solid after freeze-drying 18 h at -80 °C. Compared to previous research[1], the reaction conditions were adjusted here to suit in-situ growth in the gel, which may result in a different specific surface area of CAU-23. The yield of CAU-23 synthesized was 84%, calculated based on the mass of the organic ligand (H_2TDC).

1.2.2 Synthesis of PVA-PNIPAM

PVA-PNIPAM hydrogel was synthesized by chemical cross-linking[2-3]. A mixture containing 1.0 g NIPAM, and 500 μL 1.5 wt.% MBA was dispersed in 5 mL 10 wt.% PVA solution. Then, the mixture was slowly heated to 60 °C, subjected to magnetic stirring and sonicated for 30 mins to

eliminate air bubbles. 300 μL 4 wt.% APS and 60 μL TEMED were added to obtain the precursor, the solution was quickly transferred to the mold and polymerized at room temperature for 18 h. The products were immersed in deionized water for 24 hours to remove unreacted monomers and other chemical residues, and the final PVA-PNIPAM semi-interpenetrating gel was obtained after freeze-drying for 18 h at -80 °C (**Figure S1**). The yield of the PVA-PNIPAM hydrogel matrix after freeze-drying was 61.4%, calculated based on the total mass of the NIPAM monomer and PVA polymer.

1.2.3 Synthesis of ThermoGel-23

The metal source and ligand solutions were successively pressed into the gel by vacuum impregnation. The impregnated gel was added to CAU-23 precursor solution at concentrations of 0.5, 1.0, 1.5, and 2.0 times that of Section 1.2.1, and was refluxed and stirred for 6 h. After cooling, the hydrogel was immersed in DI for 24 h to remove unanchored CAU-23, ligands, metal salts, and other chemical residues. ThermoGel-23 was obtained after freeze-drying for 18 h at -80 °C, which was recorded as X ThermoGel-23 (X is the precursor concentration/initial CAU-23 concentration) (**Figure S6**). Other characterizations were shown in the SI. Synthesis of CAU-23-PVA-PNIPAM-sg by sol-gel method

A certain amount of CAU-23 was added to the precursor of PVA-PNIPAM, and 5 wt.%, 10 wt.%, 20 wt.%, 30 wt.%, and 40 wt.% CAU-23-PVA-PNIPAM-sg were obtained after freeze-drying (**Figure S2)**.

The sol-gel method involves adding a pre-prepared MOF to the gel precursor before monomer crosslinking to synthesize a composite gel. The scanning electron microscopy (SEM) images of the obtained CAU-23-PVA-PNIPAM-sg composites indicated that the CAU-23 particles were encapsulated within the gel matrix, significantly disrupting the originally well-defined porous channels of the PVA-PNIPAM gel (**Figure S3**). The irregular gel structure and overcoated MOF particles disfavor the diffusion of water molecules to either the polymer matrix or MOF cavities, directly leading to a decrease in the water uptake ability (**Figure S4**).

1.3 Characterizations and Measurements

The morphology of the samples was investigated by Scanning Electron Microscope (SEM, Hitachi

SU8020, Japan) equipped with Energy-Dispersive X-Ray Spectroscopy (EDS). The specific surface area and pore size distribution were investigated by Mercury Intrusion Porosimetry (MIP, Micromeritics AutoPore V 9620, USA) and Micromeritics (ASAP 2020HD88, USA). The thermal decomposition behavior of as-prepared samples was characterized by Thermogravimetric Analysis (TG, STA-449F3, Germany) and Differential Thermal Analysis (DTA) in the temperature range of 35-850 °C with heating rates 4-12 $°C·min^{-1}$. The phase structure was investigated by X-ray Diffraction (XRD, Rigaku Smartlab 9, Japan, 5° to 90° at a speed of $10°·min^{-1}$) and the Attenuated Total Reflection Fourier Transform Infrared Spectrometer (ATR-FTIR, Vertex 70, Germany, spectral resolution 2 cm^{-1}, 4000-600 cm^{-1}). The element content was investigated by Inductively Coupled Plasma Optical Emission Spectrometer (ICP-OES, Agilent 5110, USA, 12 $L·min^{-1}$ plasma flow). The hydrophilicity was investigated by Contact Angle Measuring Instrument (CA, CA200, China).

The adsorption isotherm and kinetics of samples were measured: samples were placed in a chamber (BSD-VVS&DVS, China) with a constant temperature of 25 °C and controlled relative humidity; then its mass changes were recorded by an electronic balance (Sartorius S201, Germany). Water uptake is the ratio of the sample weight increment to the original sample weight.

1.4 Two-dimensional correlation spectroscopy

Two-dimensional correlation spectroscopy (2DCS) was used to analyze the changes in the infrared spectrum of the hydrogel in the 3700-3000 cm^{-1}. 2DCS is suitable for scenarios where the interactions of polymer under external disturbances. It can effectively identify fine structures and dynamic change mechanisms[4]. The main operating steps are divided into dynamic spectrum acquisition and two-dimensional correlation spectrum analysis: i) Place the dry hydrogel in RH 80%, perform ATR-FTIR measurements every 30 minutes, and scan 20 times in total; ii) 2DCS professional software was used to analysis and export, while ORIGIN was used to draw grid diagrams.

1.5 Adsorption kinetics fitting

The adsorption kinetic properties were measured by an analytical balance (MS104TS/02, Switzerland) in a constant temperature and humidity chamber (DECCA-225L, Shenzhen), and the

mass changes were recorded and exported by the balance management software. Test conditions: The test temperature is 25 °C, the test humidity is 80%, and the adsorption equilibrium condition is 0.1 mg/60 min. Sample processing: Degassing under vacuum conditions at 80 °C for 3 hours for pretreatment to ensure complete removal of impurity gases in the sample. Input the kinetic equation through ORIGIN to fit the curve to obtain the adsorption rate (*k*) and theoretical adsorption mass (m_∞, g·g^{-1}).

$$m_t = m_\infty\left(1\text{-}e^{-k_1 t}\right) \tag{1.}$$

$$m_t = \frac{m_\infty{}^2 k_2 t}{1+m_\infty k_2 t} \tag{2.}$$

Here, m_t is the water uptake at time t, m_∞ is the fitted maximum water uptake, and k_1 and k_2 are the adsorption-rate constants of the pseudo-first-order and pseudo-second-order kinetic models, respectively.

1.6 The desorption activation energy by Kissinger equation

The desorption activation energy of the sample was measured using a thermogravimetric analyzer (STA-449F3, Germany). Test conditions: The test temperature range is 35~900 °C, the heating rate is 6, 8, 10 and 12 K·min^{-1}, the control atmosphere is nitrogen atmosphere, the crucible used is an alumina ceramic crucible, and Netzsch software is used to perform first-order analysis of the TGA data. Differentiate to obtain the DTG curve, and calculate the desorption activation energy according to the Kissinger equation. The Kissinger equation[5-6] can be used to linearly fit the DTG desorption peak temperature at different heating rates to obtain the material desorption activation energy. The equation is shown in Equation:

$$\ln\left(\frac{\beta}{\mathrm{R}T_d{}^2}\right) = -\left(\frac{E_d}{\mathrm{R}T_d}\right) - \ln\left(\frac{E_d}{K_0}\right) \tag{3.}$$

Among them, β is the heating rate, T_d is the desorption peak temperature, and E_d is the desorption activation energy.

1.7 Calculation of the accessible MOF fraction

To quantitatively evaluate the accessibility of the CAU-23 micropores within the composite, a averaging model based on CO_2 adsorption data was employed. This model assumes the total

specific surface area (SSA) of the composite is contributed additively by two parts: the accessible CAU-23 micropores and the PVA-PNIPAM matrix, the calculation is:

$$SSA_{\text{ThermoGel-23, CO}_2} = SSA_{\text{CAU-23, CO}_2} \times \omega_{\text{CAU-23}} \times F_{\text{Accessible MOF}} + SSA_{\text{PVA-PNIPAM, CO}_2} \times \omega_{\text{PVA-PNIPAM}} \quad (4.)$$

Among them, $SSA_{\text{ThermoGel-23, CO}_2}$ is the specific surface area (SSA) of the ThermoGel-23 by CO_2 adsorption, $SSA_{\text{CAU-23, CO}_2}$ is the SSA of pure CAU-23 powder, $SSA_{\text{PVA-PNIPAM, CO}_2}$ is the SSA of PVA-PNIPAM hydrogel matrix, $\omega_{\text{CAU-23}}$ is the mass fraction of CAU-23 in ThermoGel-23 (determined from elemental analysis and TGA, see main text and **Table S2**), $\omega_{\text{PVA-PNIPAM}}$ is the mass fraction of PVA-PNIPAM, and $F_{\text{Accessible MOF}}$ is the Accessible MOF Fraction, representing the portion of CAU-23 micropores within the composite that are effectively accessible to the CO_2 probe molecules. The calculated Accessible MOF Fraction ($F_{Accessible\ MOF}$) is approximately 43.9%.

2. Supporting Tables

Table S1. Raw materials and Chemical reagents information

Chemical reagents	Formula	Specification	Manufacturer
Deionized water	H_2O	18.2 MΩ·cm	Millpore Milli-Q
Methyl alcohol	CH_3OH	AR	Macklin, China
Poly(vinyl alcohol)-1799	$[C_2H_4O]_n$	98～99%	Macklin, China
N-isopropylacrylamide	$C_6H_{11}NO$	98%	Macklin, China
N,N'-methylenebisacrylamide	$C_7H_{10}N_2O_2$	99%	Macklin, China
Ammonium persulfate	$H_8N_2O_8S_2$	98.5%	Boer, China
N,N,N',N'-tetramethylethylenediamine	$C_6H_{16}N_2$,	99%	Macklin, China
2,5-thiophenedicarboxylic acid	$C_6H_4O_4S$	98%	Macklin, China
Sodium hydroxide	NaOH	95%	DaMao, China
Sodium aluminate	$NaAlO_2$	AR	Macklin, China
Aluminum chloride hexahydrate	$AlCl_3H_{12}O_6$	97%	Macklin, China
Air	$20\%O_2/N_2$	99.9%	HuaYuan, China
Nitrogen	N_2	99.9%	HuaYuan, China
Liquid nitrogen	N_2	>99.9%	JunFang, China

Table S2. Sample precursor metal ion and ligand concentration

Samples	CAU-23 precursor concentration (M)			NaCl (%)	TG residues (%)	CAU-23 Loading (%)	
	Na_2TDC	$AlCl_3$	$NaAlO_2$				
0.5 ThermoGel-23	0.095	0.072	0.024	0.01 [a]	2.00	8.48 [a]	8.37 [b]
1.0 ThermoGel-23	0.190	0.143	0.048	2.93 [a]	4.30	15.6 [a]	5.76 [b]
1.5 ThermoGel-23	0.285	0.215	0.072	9.01 [a]	15.22	62.5 [a]	26.1 [b]
2.0 ThermoGel-23	0.380	0.286	0.096	9.54 [a]	22.93	63.2 [a]	56.3 [b]

[a] the mass fractions of NaCl and CAU-23 were determined by ICP-OES measurement of Na and Al content.

[b] the mass fraction of CAU-23 was calculated based on the difference between the amount of

NaCl and the thermogravimetric residue, which is taken as the Al_2O_3 pyrolysis product of CAU-23.

Table S3. Specific surface area, pore volume and diameter of composite gels

Sample	S ($m^2 \cdot g^{-1}$)	Pore volume ($cc \cdot g^{-1}$)	Pore diameter (nm)	R^2
PVA-PNIPAM	22.21	0.01	1.85	0.995
CAU-23	1615.06	0.58	1.00	0.999
ThermoGel-23	407.89	0.17	1.17	0.998

Table S4. Thermal decomposition temperature of hydrogel by the tangent extrapolation method

Sample	k_{min} ($\% \cdot min^{-1}$)	T_{min} [a] (°C)	k_{max} ($\% \cdot min^{-1}$)	T_{max} [b] (℃)	T_d [c] (°C)
PVA-PNIPAM	-0.3	234.6	-13.9	411.4	411.1
CAU-23	-0.6	371.5	-11.7	459.8	461.4
0.5 ThermoGel-23	0.0	202.7	-10.6	384.2	379.2
1.0 ThermoGel-23	-0.1	201.0	-13.1	400.6	397.8
1.5 ThermoGel-23	0.0	200.3	-5.3	383.2	377.0
2.0 ThermoGel-23	-0.1	203.6	-10.4	399.1	396.1

[a] Slowest decomposition temperature. [b] Fastest decomposition temperature. [c] Thermal decomposition temperature.

Table S5 Hydrogel adsorption pseudo-first-order and pseudo-second-order kinetic model equation parameters

Sample		Pseudo-first-order kinetics			Pseudo-second-order kinetics		
	m [a]	m_∞ [b]	k_1 (min^{-1})	R^2	m_∞ [b]	k_2 ($g \cdot g^{-1} \cdot min^{-1}$)	R^2
PVA-PNIPAM	0.159	0.145	0.011	0.885	0.158	0.102	0.967
CAU-23	0.297	0.288	1.182	0.989	0.292	10.813	0.991
ThermoGel-23	0.649	0.637	0.049	0.995	0.663	0.146	0.972

[a] Maximum adsorption capacity measured ($g \cdot g^{-1}$). [b] Kinetic fitting maximum adsorption capacity ($g \cdot g^{-1}$).

Table S6 Details of the weight loss stage of hydrogel

Sample	Stage	weight loss
CAU-23	I	water loss (surface water, internal water, capillary condensate)
	II	uncoordinated ligand loss
	III	CAU-23 collapse and thermal decomposition
PVA-PNIPAM	I	water loss (surface water, internal water)
	II	polymer chain decomposes
ThermoGel-23	I	water loss (surface water, internal water, capillary condensate)
	II	polymer chain decomposes, uncoordinated ligand loss
	III	CAU-23 collapse and thermal decomposes

Table S7 Desorption peak temperatures (T_d) and desorption activated energies (E_d) of hydrogel with different heating rates

Sample	T_d (°C) at different β (°C min^{-1})					E_d ($kJ \cdot mol^{-1}$)	R^2
	4	6	8	10	12		
$CaCl_2$-PVA-PNIPAM	-	67.75	74.76	78.82	84.00	38.1	0.994
ThermoGel-23	56.49	64.97	71.64	81.04	84.66	31.0	0.982

Table S8 The apparent desorption enthalpies of hydrogels during water escape by differential scanning calorimetry (DSC).

Sample	E_d ($kJ \cdot mol^{-1}$)
CAU-23	23.5
PVA-PNIPAM	31.5
ThermoGel-23	24.9
ThermoGel-23 without NaCl	27.5

Table S9 MOF structural parameters and hygroscopic properties

MOF	Metal	S_{BET}	Solvent	Water uptake	Adsorb RH (%)	Ref.

		$(m^2 \cdot g^{-1})$		$(g \cdot g^{-1})$	Start	End	
CUK-1	Co	510	H_2O	0.28	-	12	[7]
CUK-1	Ni	520	H_2O	0.3	-	12	[7]
CUK-1	Mg	580	H_2O	0.36	23	28	[7]
CAU-10	Al	635	EA	0.36	15	25	[8]
CAU-23	Al	1250	H_2O	0.37	-	30	[1]
MIP-200	Zr	1000	MT	0.46	-	17	[9]
MIL-125-NH_2	Ti	1553	DMF	0.4	-	20	[10]
ZJNU-30	Zr	3116	DMA	1.2	-	21	[11]
MOF-303	Al	-	H_2O	0.48	10	25	[12]
MOF-801-SC	Zr	990	DMF/MT	0.36	5	15	[13]
MOF-841	Zr	1390	DMF/FA	0.5	-	26	[14]
MOF-808	Zr	2360	DMF/FA	0.6	28	32	[14]
Ni-TPP	Ni	1975	DMF/EA	0.84	26	64	[15]
Ni-BPP	Ni	2039	DMF/EA	0.72	8	32	[15]
UiO-66	Zr	1290	DMF	0.44	30	35	[16]
MIL-100	Fe	1917	DMF	0.77	25	45	[17]
MIL-100	Al	1814	HNO_3	0.5	25	45	[17]
BIT-66	V	1417	H_2O	0.71	-	60	[18]
Cr-soc-MOF-1	Cr	4549	DMF	1.95	42	60	[19]
Y-shp-MOF-5	Y	1550	DMF	0.45	55	72	[20]
PIZOF-2	Zr	1250	DMF	0.68	70	74	[21]
NU-1500	Cr	3580	DMF	1.09	-	90	[22]
MIL-160/PAA-NH_2	Al	782	H_2O	0.25	-	15	[23]
Ni-MOF/PDA	Ni	1661	DMF	1.03	35	95	[24]
PNIPAM/MIL-101	Cr	2200	H_2O	1.15	40	90	[25]
MIL-101@xerogel	Cr	2530	DMF	0.89	35	50	[26]

DMF-N,N-Dimethylformamide, DMA-N,N-Dimethylaniline, MT-Methyl alcohol, EA-Ethyl alcohol, FA-Formic acid.

3. Supporting Figures

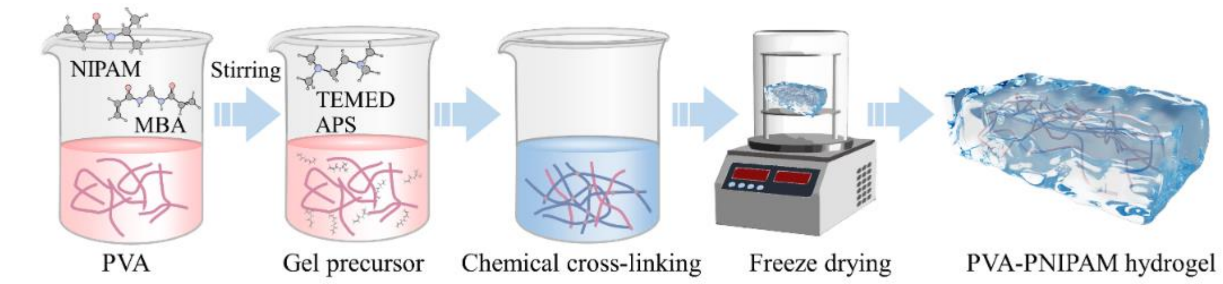

Figure S1 Synthesis of PVA-PNIPAM by chemical cross-linking.

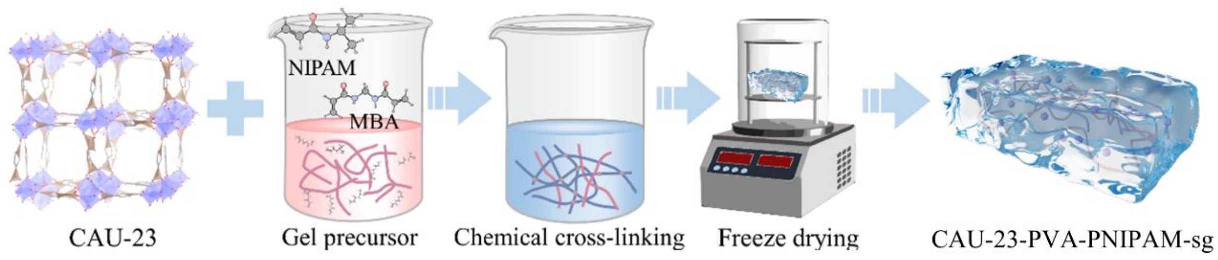

Figure S2 Synthesis of CAU-23-PVA-PNIPAM-sg by sol-gel method without interface control.

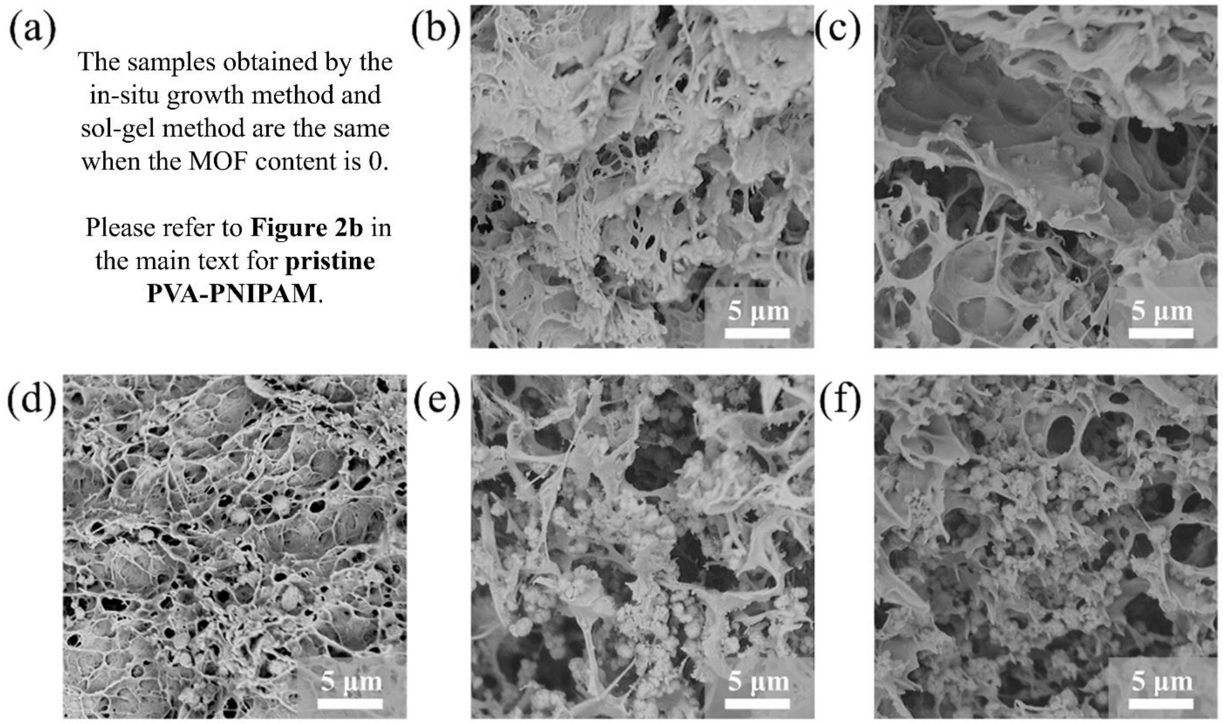

Figure S3 SEM images of CAU-23-PVA-PNIPAM-sg in sol-gel method: (a) PVA-PNIPAM (the samples obtained by *in-situ* growth method and sol-gel method are the same when the MOF content is 0.), (b) 5 wt.% CAU-23-PVA-PNIPAM-sg, (c) 10 wt.% CAU-23-PVA-PNIPAM-sg, (d) 20 wt.% CAU-23-PVA-PNIPAM-sg, (e) 30 wt.% CAU-23-PVA-PNIPAM-sg, (f) 40 wt.% CAU-23-PVA-PNIPAM-sg.

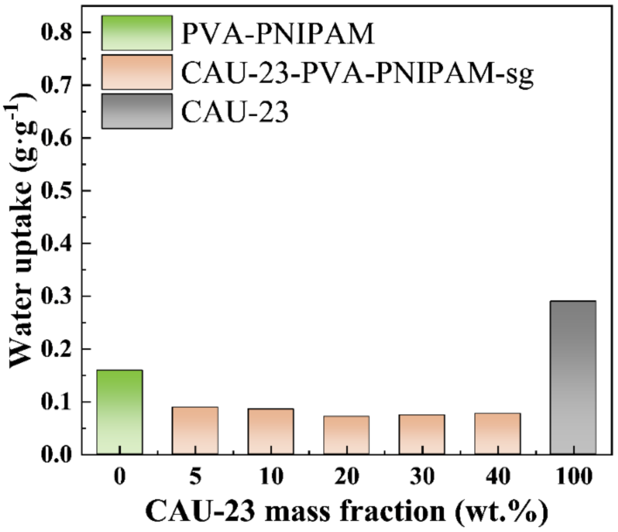

Figure S4 CAU-23-PVA-PNIPAM-sg water uptake with different CAU-23 mass fractions by sol-gel method (T=25 °C, RH 80%).

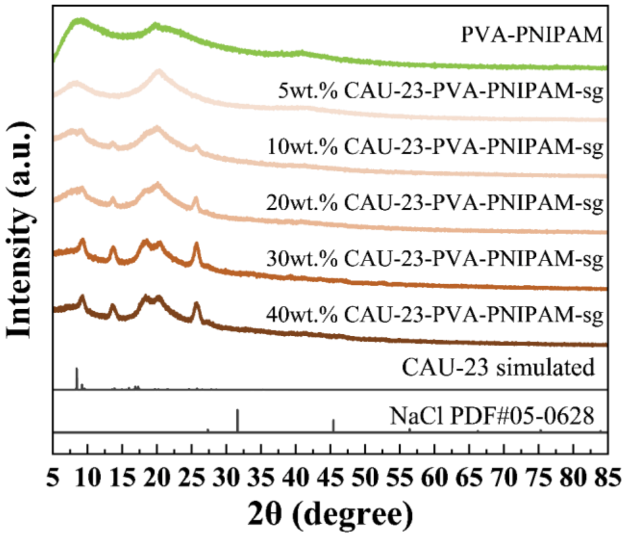

Figure S5 CAU-23-PVA-PNIPAM-sg prepared by sol-gel method with different CAU-23 mass fractions.

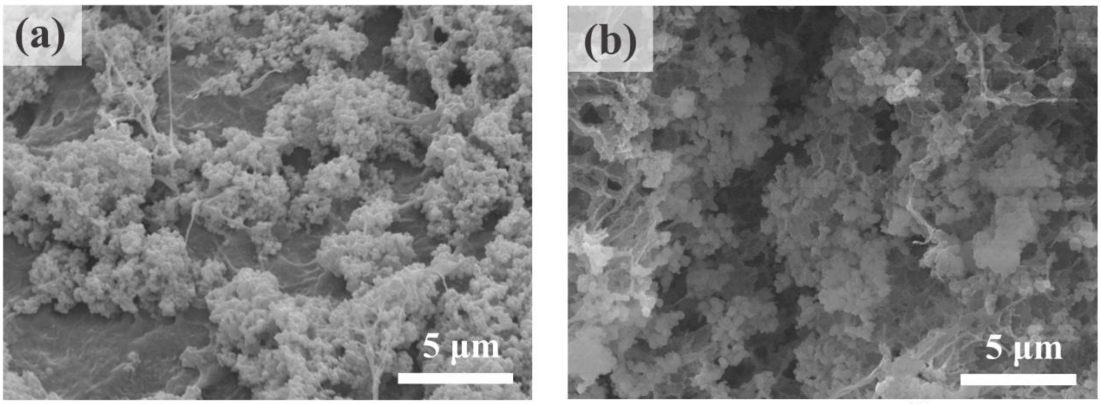

Figure S6 (a) SEM image of ThermoGel-23 surface. (b) SEM image of the internal cross-section of ThermoGel-23.

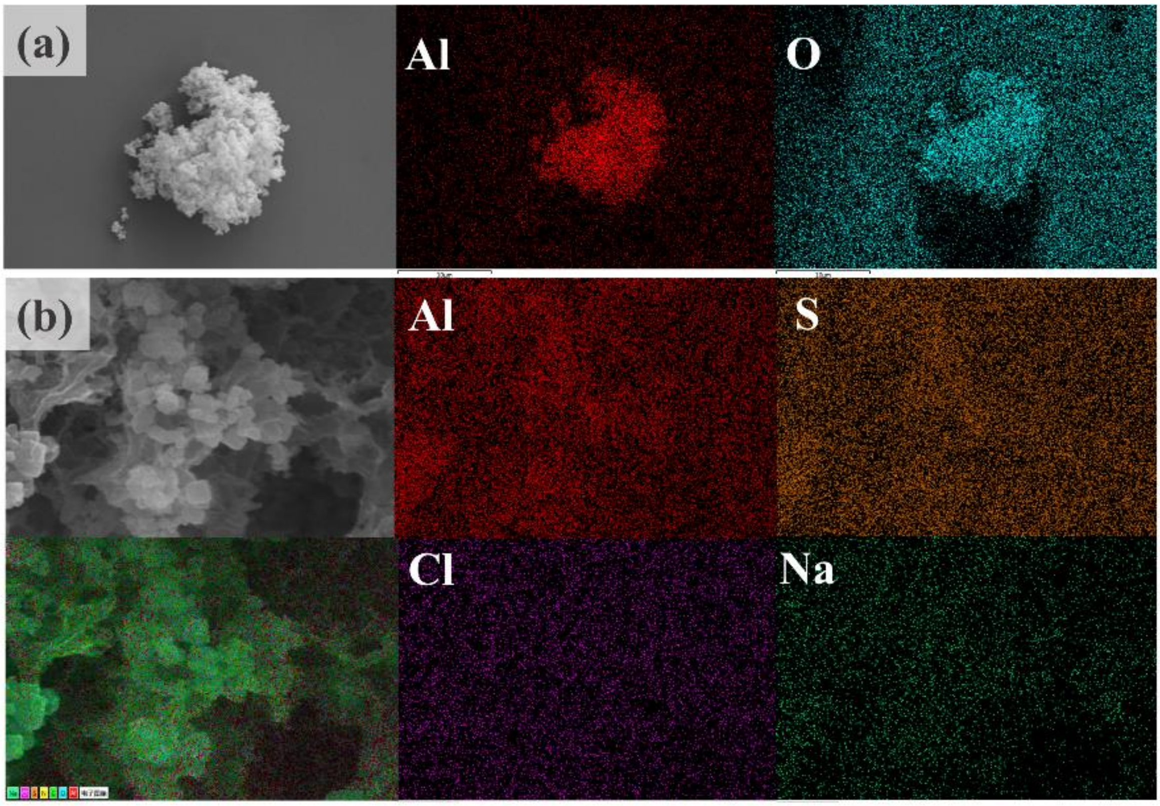

Figure S7 (a) Energy spectrum images of CAU-23. (b) Energy spectrum images of ThermoGel-23.

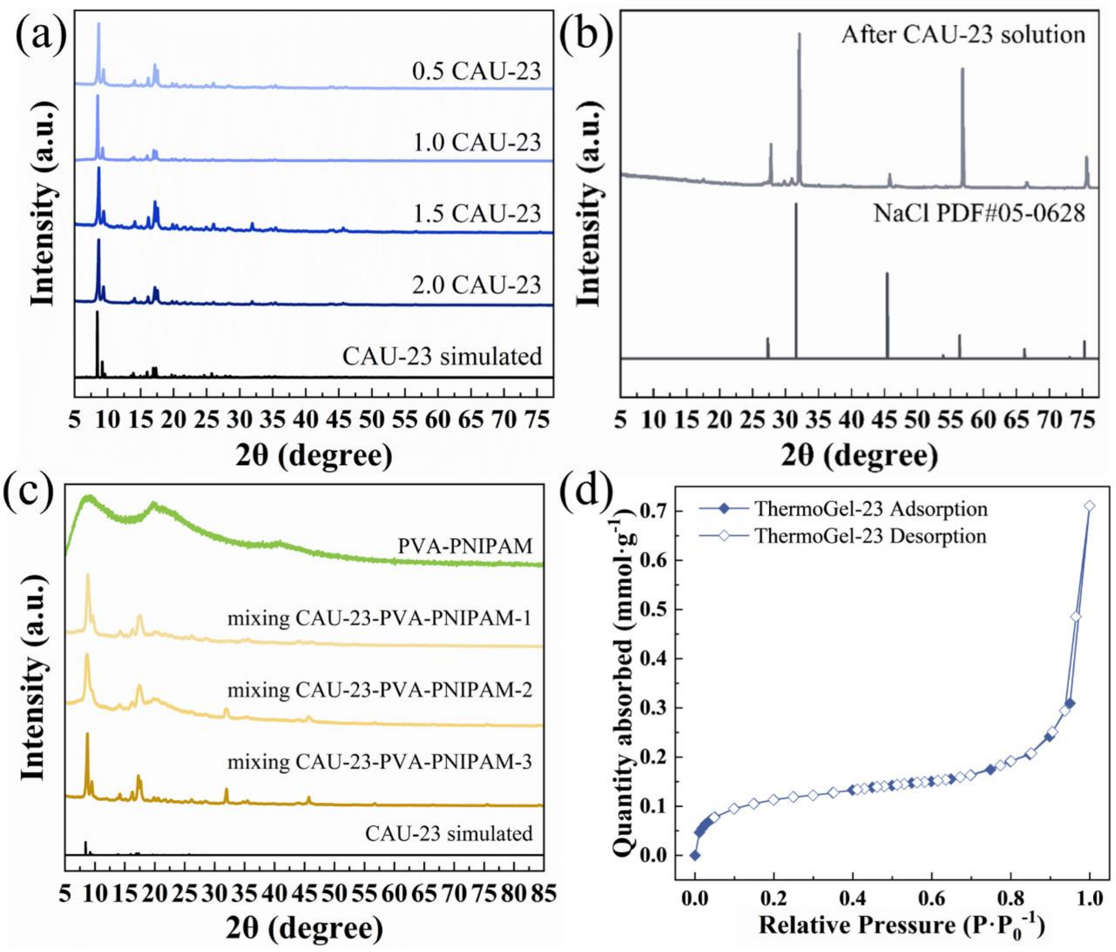

Figure S8 (a) XRD patterns of CAU-23 synthesized from precursor liquids with different concentrations. (b) Crystallization of the supernatant after CAU-23 reflux. (c) ThermoGel-23 physically mixed with different mass ratios. (d) N_2 adsorption and desorption curve.

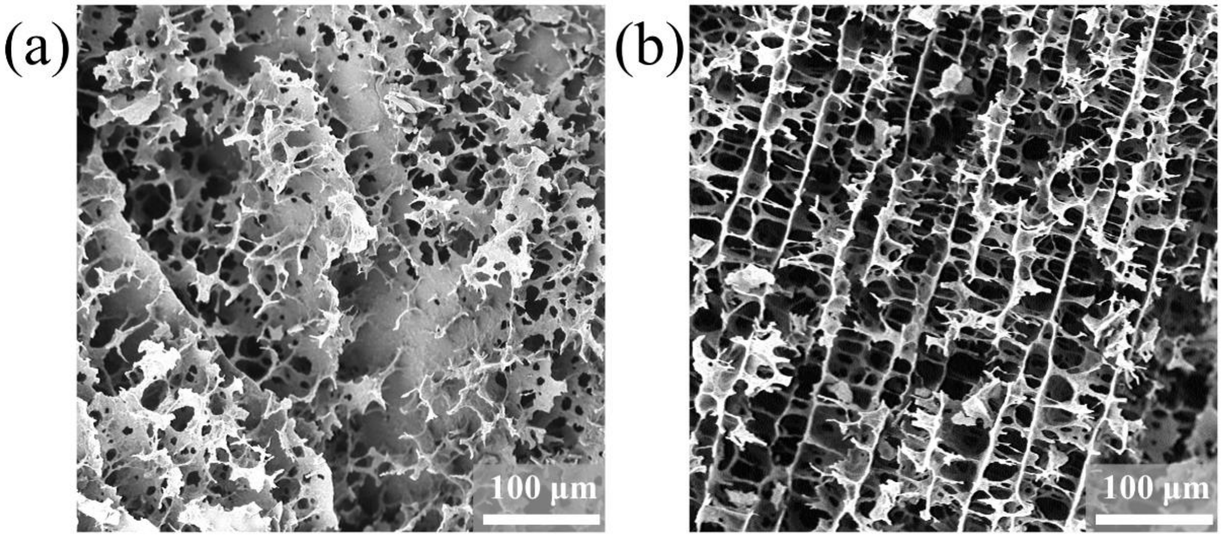

Figure S9 SEM images of PNIPAM. (a) Section view. (b) Plan view.

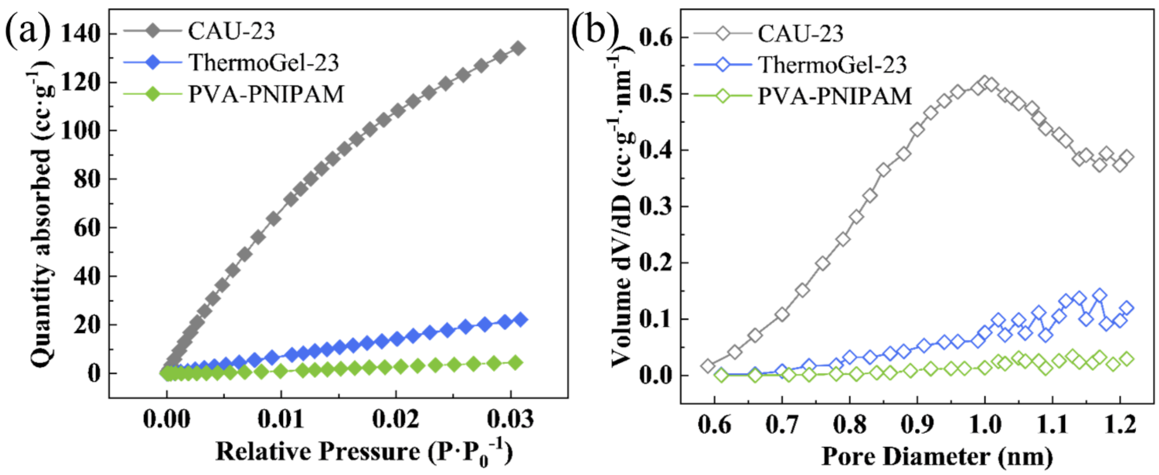

Figure S10 (a) Carbon dioxide adsorption curves for micropore analysis. (b) pore size distribution.

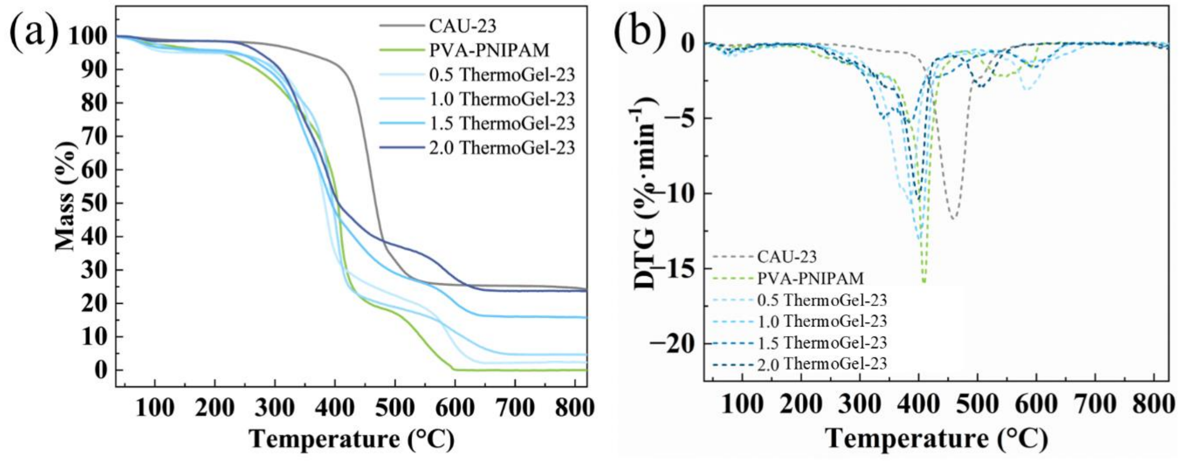

Figure S11 (a) TG and (b) DTG curves of hydrogels.

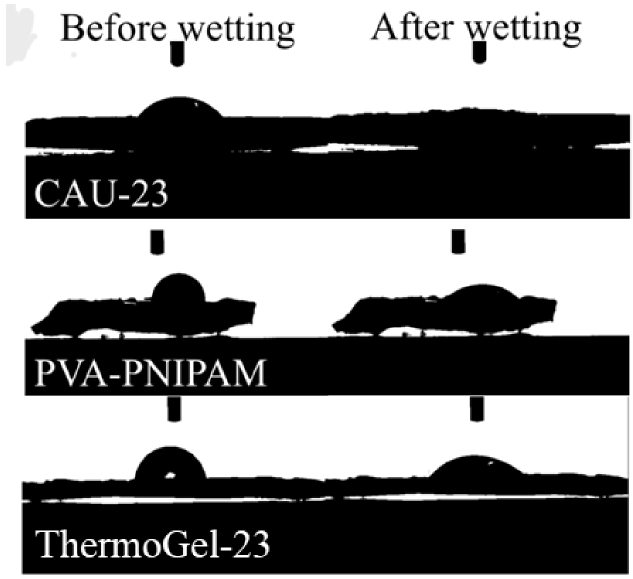

Figure S12 water contact angle and wetting optical images.

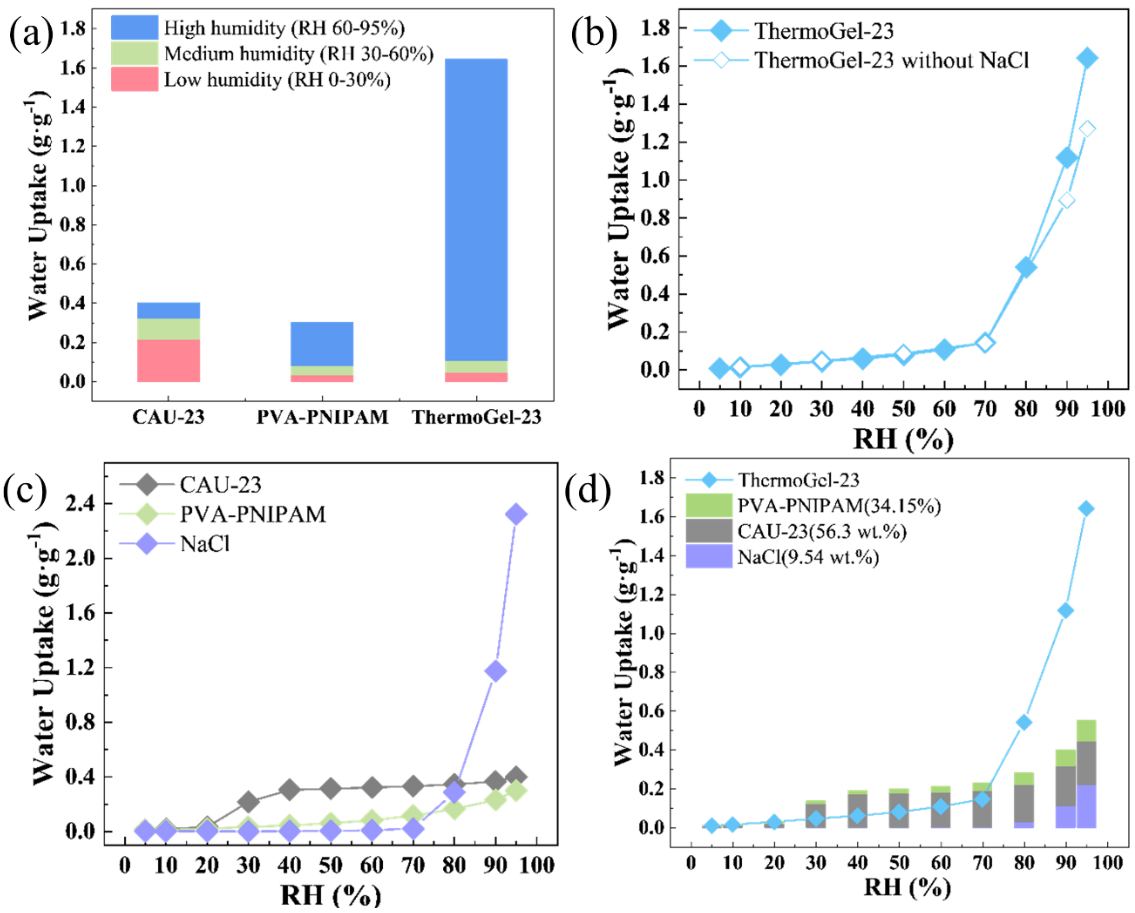

Figure S13 (a) Water uptake of hydrogels across different humidity ranges. (b) Water vapor adsorption isotherms of ThermoGel-23 without NaCl via thorough washing (T= 25 °C). (c) Water vapor adsorption isotherms of components (T= 25 °C). (d) Stacked bar graphs of the theoretical water uptake contribution of components (T= 25 °C).

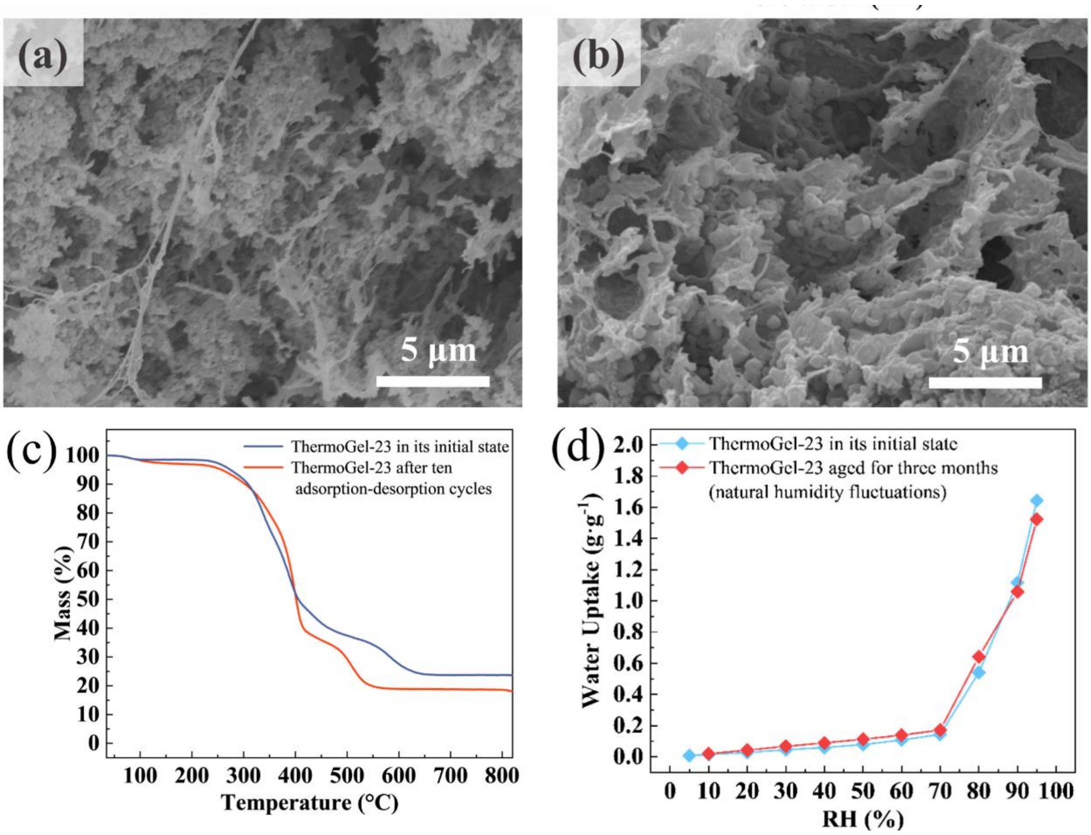

Figure S14 (a) SEM images of ThermoGel-23 in its initial state. (b) SEM images of ThermoGel-23 after ten adsorption-desorption cycles. (c) TG curves of in its initial state and after ten adsorption-desorption cycles (d) Water vapor adsorption-desorption isotherms of ThermoGel-23 in its initial state and after three months of aging (natural humidity fluctuations).

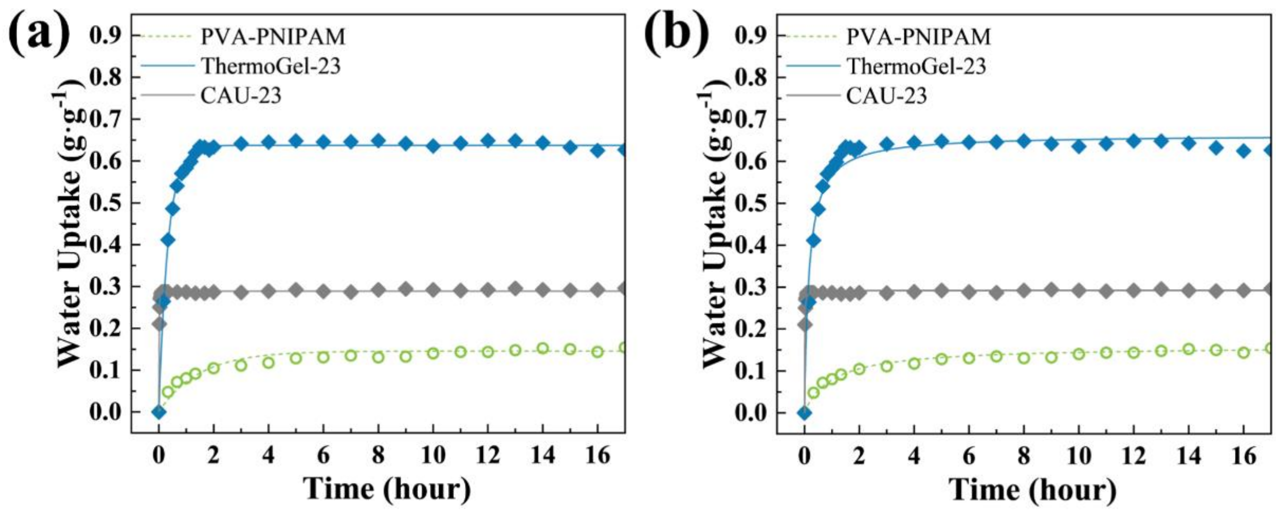

Figure S15 Hydrogel adsorption kinetic equation fitting curves. (a) pseudo-first-order, (b) pseudo-second-order

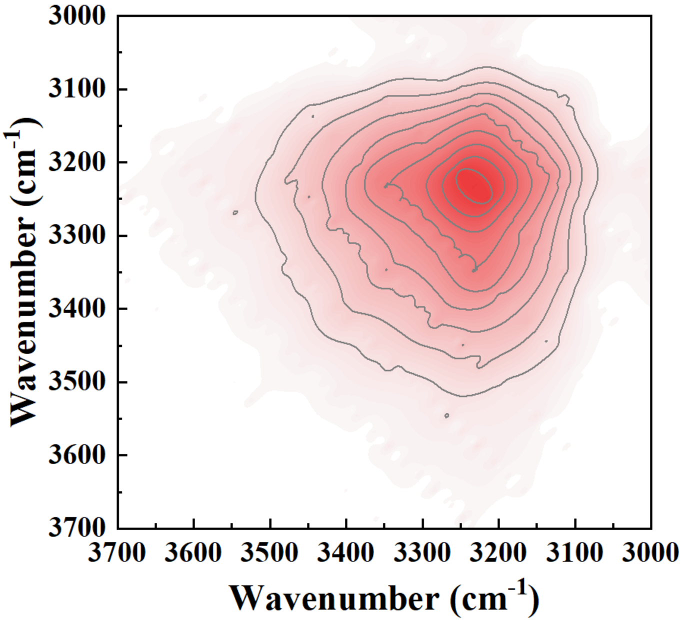

Figure S16 The 2D-FTIR correlation synchronous spectra of ThermoGel-23 (3700-3000 cm^{-1})

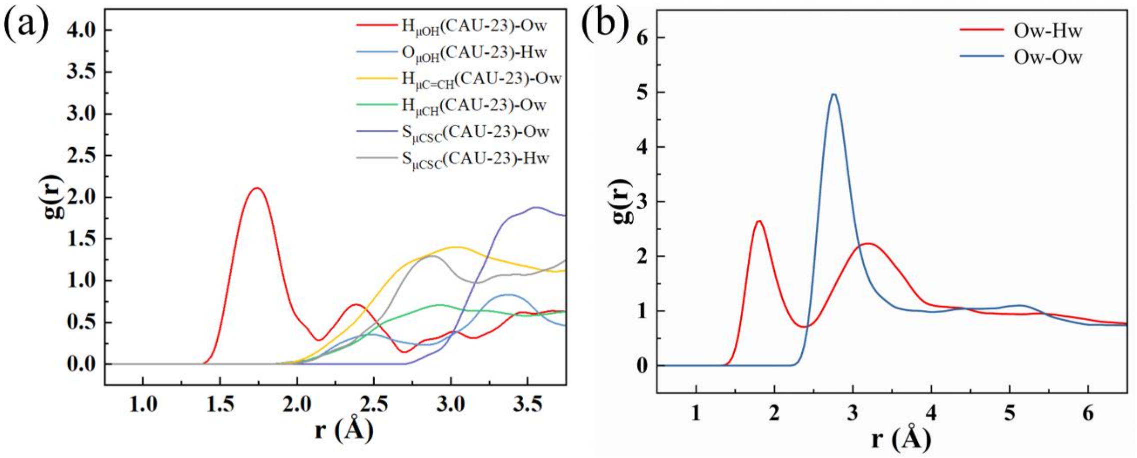

Figure S17 (a) Radial distribution functions of the water network. (b) Radial distribution functions between atoms of H_2O and CAU-23.

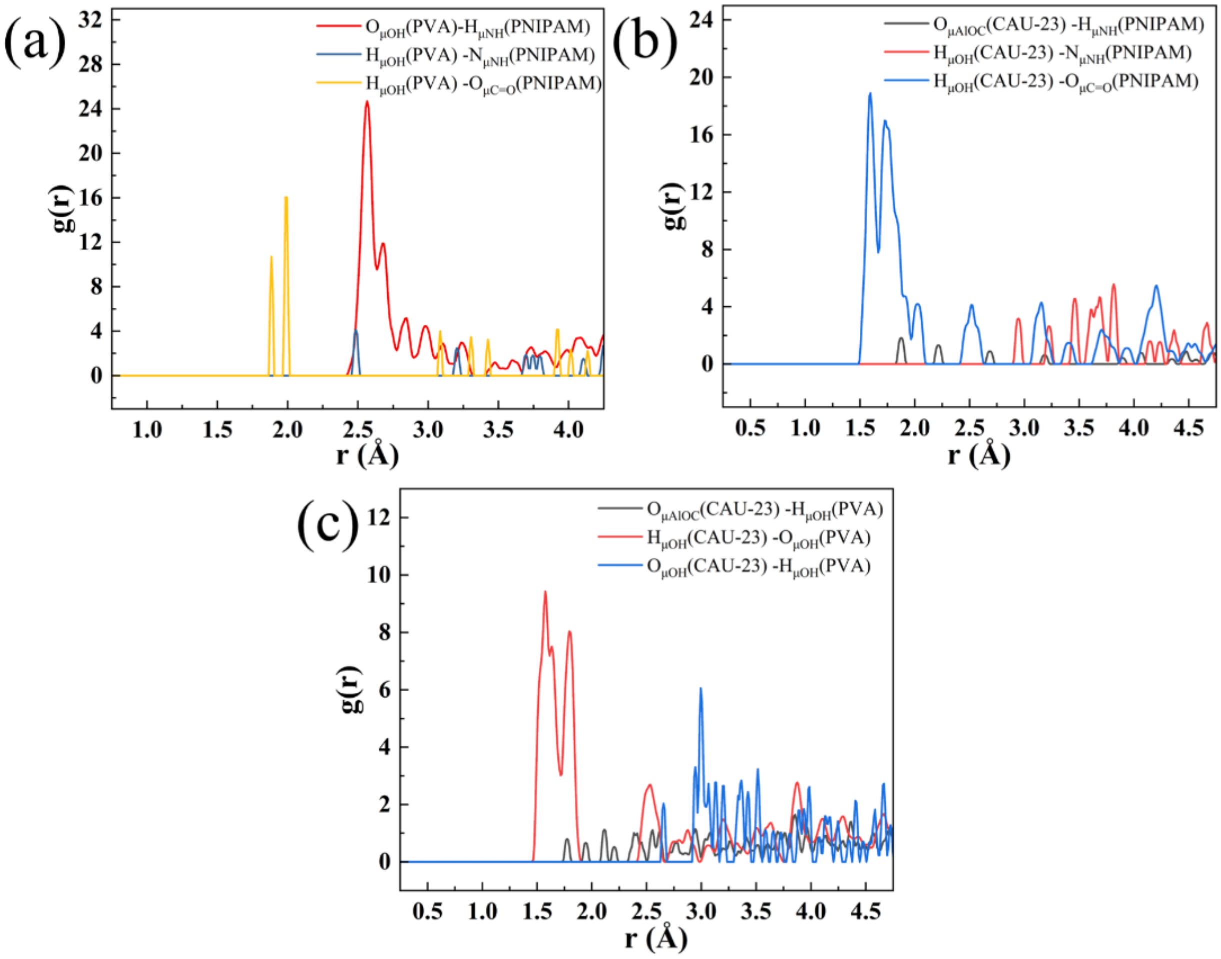

Figure S18 (a) radial distribution functions between atoms of PVA and PNIPAM. (b) radial distribution functions between atoms of CAU-23 and PNIPAM And (c) radial distribution functions between atoms of CAU-23 and PVA.

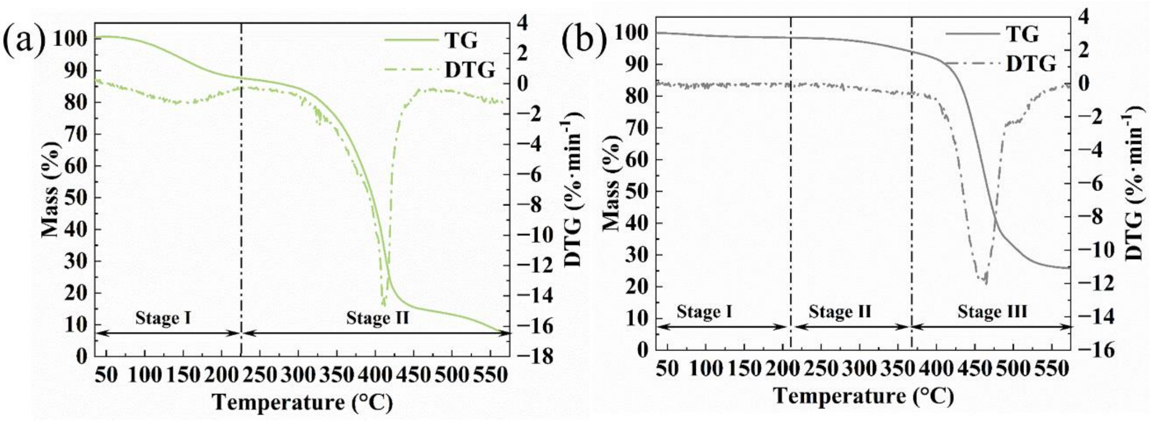

Figure S19 (a) The TG and DTG curve of PVA-PNIPAM, (b) The TG and DTG curve of CAU-23

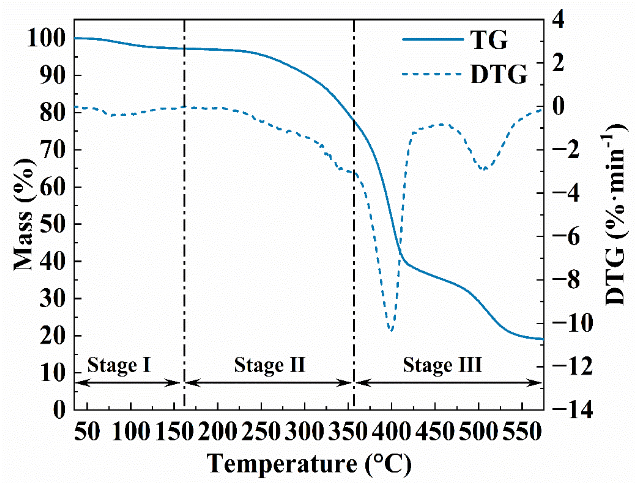

Figure 1

Figure S20 The TG and DTG curve of ThermoGel-23

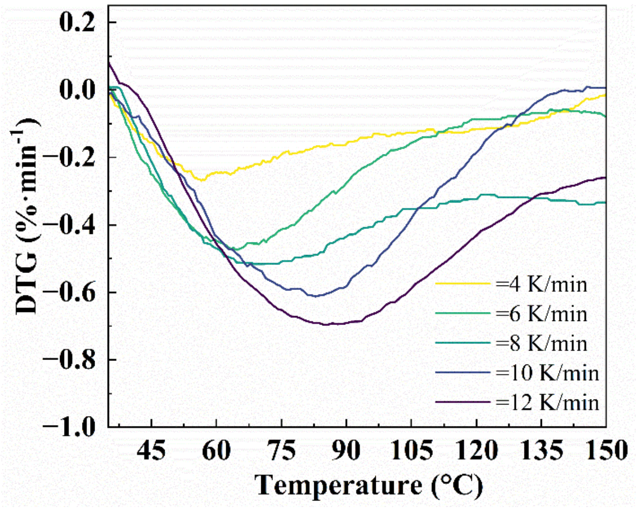

Figure S21 ThermoGel-23 desorption activation energy Kissinger equation fitting results.

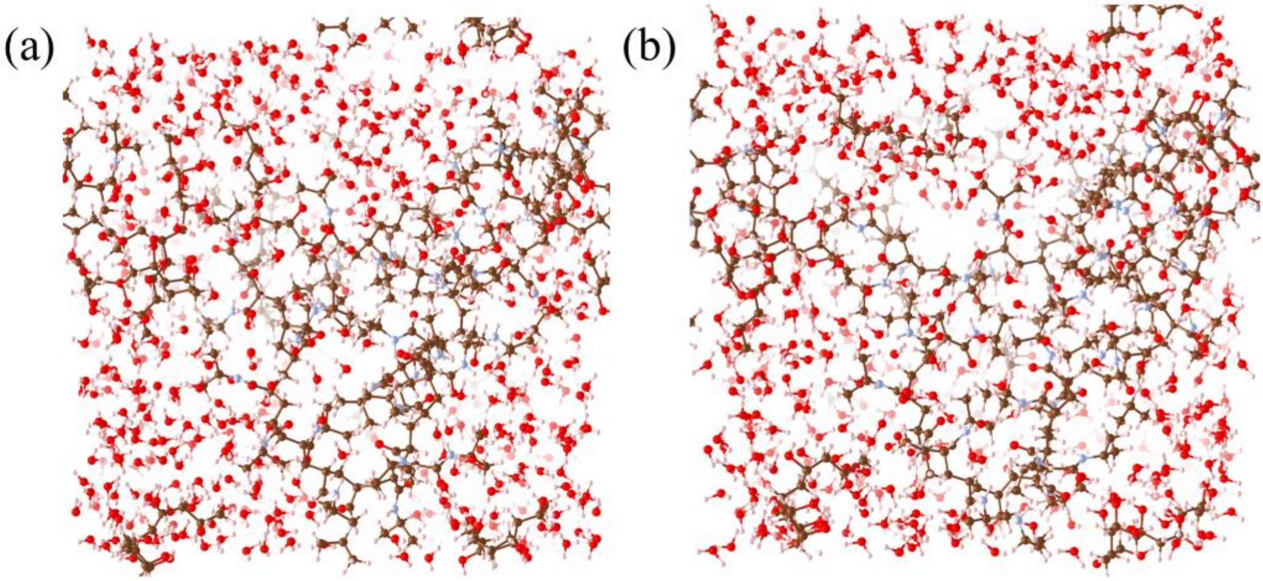

1

2 **Figure S22** Swelling model of ThermoGel-23 gel polymer chain after molecular dynamics
3 calculation. (a) PNIPAM-α (T< LCST), (b) PNIPAM-β (T > LCST).

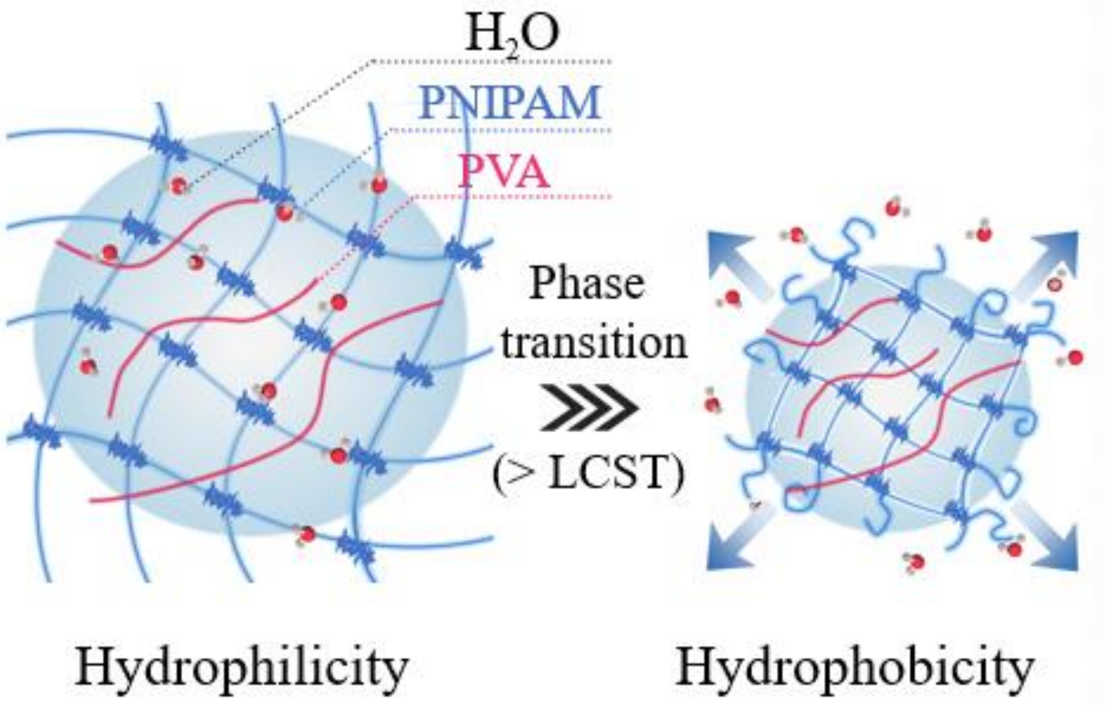

4

5 **Figure S23** Schematic diagram of polymer network deswelling.

6

4. References

1. D. Lenzen, J. J. Zhao, S. J. Ernst, M. Wahiduzzaman, A. Ken Inge, D. Fröhlich, H. Xu, H.-J. Bart, C. Janiak, S. Henninger, G. Maurin, X. D. Zou, N. Stock, "A Metal–Organic Framework for Efficient Water-Based Ultra-Low-Temperature-Driven Cooling" *Nature Communications* (2019): 3025,
https://doi.org/10.1038/s41467-019-10960-0
2. Y. Cai, W. Shen, S. Loo, W. B. Krantz, R. Wang, A. G. Fane, X. Hu, "Towards Temperature Driven Forward Osmosis Desalination Using Semi-IPN Hydrogels as Reversible Draw Agents" *Water Research* (2013): 3773,
https://doi.org/10.1016/j.watres.2013.04.034
3. H. Zhang, J. Liu, F. Shi, T. Li, H. Zhang, D. Yang, Y. Li, Z. Tian, N. Zhou, "A Novel Bidirectional Fast Self-Responsive PVA-PNIPAM/$Li_mCs_nWo_3$ Composite Hydrogel for Smart Window Applications" *Chemical Engineering Journal* (2022): 133353,
https://doi.org/10.1016/j.cej.2021.133353
4. L. Hou, P.-Y. Wu, "Applications of Two-Dimensional Correlation Infrared Spectroscopy in the Characterization of Polymers" *Acta Polymerica Sinica* (2022): 522,
https://doi.org/10.11777/j.issn1000-3304.2021.21362
5. A. Khutia, H. U. Rammelberg, T. Schmidt, S. Henninger, C. Janiak, "Water Sorption Cycle Measurements on Functionalized MIL-101Cr for Heat Transformation Application" *Chemistry of Materials* (2013): 790,
https://doi.org/10.1021/cm304055k
6. M. B. H. Othman, A. Khan, Z. Ahmad, M. R. Zakaria, F. Ullah, H. M. Akil, "Kinetic Investigation and Lifetime Prediction of Cs–NIPAM–MBA-Based Thermo-Responsive Hydrogels" *Carbohydrate Polymers* (2016): 1182,
https://doi.org/10.1016/j.carbpol.2015.10.034
7. J. S. Lee, J. W. Yoon, P. G. Mileo, K. H. Cho, J. Park, K. Kim, H. Kim, M. F. De Lange, F. Kapteijn, G. Maurin, "Porous Metal–Organic Framework CUK-1 for Adsorption Heat Allocation toward Green Applications of Natural Refrigerant Water" *ACS Applied Materials & Interfaces* (2019): 25778,
https://doi.org/10.1021/acsami.9b02605
8. D. Lenzen, P. Bendix, H. Reinsch, D. Frohlich, H. Kummer, M. Mollers, P. P. C. Hugenell, R. Glaser, S. Henninger, N. Stock, "Scalable Green Synthesis and Full-Scale Test of the Metal-Organic Framework CAU-10-H for Use in Adsorption-Driven Chillers" *Advanced Materials* (2018): 1705869,
https://doi.org/10.1002/adma.201705869
9. S. Wang, J. S. Lee, M. Wahiduzzaman, J. Park, M. Muschi, C. Martineau-Corcos, A. Tissot, K. H. Cho, J. Marrot, W. Shepard, G. Maurin, J. Chang, C. Serre, "A Robust Large-Pore Zirconium Carboxylate Metal-Organic Framework for Energy-Efficient Water-Sorption-Driven Refrigeration" *Nature Energy* (2018): 985,
https://doi.org/10.1038/s41560-018-0261-6
10. M. Sohail, Y. Yun, E. Lee, S. K. Kim, K. Cho, J. Kim, T. W. Kim, J. Moon, H. Kim, "Synthesis of Highly Crystalline NH_2-Mil-125 (Ti) with S-Shaped Water Isotherms for Adsorption Heat

Transformation" *Crystal Growth & Design* (2017): 1208, https://doi.org/10.1021/acs.cgd.6b01597

11. H. Liu, F. Chen, D. Bai, J. Jiao, W. Zhou, T. Yildirim, Y. He, "High-Pressure Methane Adsorption in Two Isoreticular Zr-Based Metal–Organic Frameworks Constructed from C3-Symmetrical Tricarboxylates" *Crystal Growth & Design* (2017): 248, https://doi.org/10.1021/acs.cgd.6b01507
12. F. Fathieh, M. J. Kalmutzki, E. A. Kapustin, P. J. Waller, J. Yang, O. M. Yaghi, "Practical Water Production from Desert Air" *Science Advances* (2018): eaat3198, https://doi.org/doi:10.1126/sciadv.aat3198
13. H. Kim, S. Yang, S. R. Rao, S. Narayanan, E. A. Kapustin, H. Furukawa, A. S. Umans, O. M. Yaghi, E. N. Wang, "Water Harvesting from Air with Metal-Organic Frameworks Powered by Natural Sunlight" *Science* (2017): 430, https://doi.org/10.1126/science.aam8743
14. H. Furukawa, F. Gándara, Y.-B. Zhang, J. Jiang, W. L. Queen, M. R. Hudson, O. M. Yaghi, "Water Adsorption in Porous Metal–Organic Frameworks and Related Materials" *Journal of the American Chemical Society* (2014): 4369, https://doi.org/10.1021/ja500330a
15. J. Zheng, R. S. Vemuri, L. Estevez, P. K. Koech, T. Varga, D. M. Camaioni, T. A. Blake, B. P. Mcgrail, R. K. Motkuri, "Pore-Engineered Metal–Organic Frameworks with Excellent Adsorption of Water and Fluorocarbon Refrigerant for Cooling Applications" *Journal of the American Chemical Society* (2017): 10601, https://doi.org/10.1021/jacs.7b04872
16. G. E. Cmarik, M. Kim, S. M. Cohen, K. S. Walton, "Tuning the Adsorption Properties of UiO-66 Via Ligand Functionalization" *Langmuir* (2012): 15606, https://doi.org/10.1021/la3035352
17. F. Jeremias, A. Khutia, S. K. Henninger, C. Janiak, "MIL-100 (Al, Fe) as Water Adsorbents for Heat Transformation Purposes—a Promising Application" *Journal of Materials Chemistry* (2012): 10148, https://doi.org/10.1039/c2jm15615f
18. D. Ma, P. Li, X. Duan, J. Li, P. Shao, Z. Lang, L. Bao, Y. Zhang, Z. Lin, B. Wang, "A Hydrolytically Stable Vanadium(IV) Metal-Organic Framework with Photocatalytic Bacteriostatic Activity for Autonomous Indoor Humidity Control" *Angewandte Chemie International Edition* (2020): 3905, https://doi.org/10.1002/anie.201914762
19. S. M. Towsif Abtab, D. Alezi, P. M. Bhatt, A. Shkurenko, Y. Belmabkhout, H. Aggarwal, Ł. J. Weseliński, N. Alsadun, U. Samin, M. N. Hedhili, M. Eddaoudi, "Reticular Chemistry in Action: A Hydrolytically Stable MOF Capturing Twice Its Weight in Adsorbed Water" *Chem* (2018): 94, https://doi.org/10.1016/j.chempr.2017.11.005
20. R. G. Abdulhalim, P. M. Bhatt, Y. Belmabkhout, A. Shkurenko, K. Adil, L. J. Barbour, M. Eddaoudi, "A Fine-Tuned Metal–Organic Framework for Autonomous Indoor Moisture Control" *Journal of the American Chemical Society* (2017), https://doi.org/10.1021/jacs.7b04132

21. A. Schaate, P. Roy, T. Preuße, S. J. Lohmeier, A. Godt, P. Behrens, "Porous Interpenetrated Zirconium–Organic Frameworks (PIZOFs): A Chemically Versatile Family of Metal–Organic Frameworks" *Chemistry – A European Journal* (2011): 9320, https://doi.org/10.1002/chem.201101015

22. Z. Chen, P. Li, X. Zhang, P. Li, M. C. Wasson, T. Islamoglu, J. F. Stoddart, O. K. Farha, "Reticular Access to Highly Porous acs-MOFs with Rigid Trigonal Prismatic Linkers for Water Sorption" *Journal of the American Chemical Society* (2019): 2900, https://doi.org/10.1021/jacs.8b13710

23. T. Li, H. Yu, J. Mi, C. Li, H. Meng, J. Jin, "Highly Hydrophilic Acrylate Copolymer Supported MIL-160 for Air Water Harvesting" *Chemical Physics Letters* (2023): 140391, https://doi.org/10.1016/j.cplett.2023.140391

24. L. Peng, S. Yang, D. T. Sun, M. Asgari, W. L. Queen, "MOF/Polymer Composite Synthesized Using a Double Solvent Method Offers Enhanced Water and CO_2 Adsorption Properties" *Chemical Communications* (2018): 10602, https://doi.org/10.1039/C8CC05428B

25. A. Karmakar, P. G. M. Mileo, I. Bok, S. B. Peh, J. Zhang, H. Yuan, G. Maurin, D. Zhao, "Thermo-Responsive MOF/Polymer Composites for Temperature-Mediated Water Capture and Release" *Angewandte Chemie International Edition* (2020): 11003, https://doi.org/10.1002/anie.202002384

26. M. Wickenheisser, A. Herbst, R. Tannert, B. Milow, C. Janiak, "Hierarchical MOF-Xerogel Monolith Composites from Embedding MIL-100(Fe,Cr) and MIL-101(Cr) in Resorcinol-Formaldehyde Xerogels for Water Adsorption Applications" *Microporous and Mesoporous Materials* (2015): 143, https://doi.org/10.1016/j.micromeso.2015.05.017
